# Complex Genetic Interactions in a Quantitative Trait Locus

**DOI:** 10.1371/journal.pgen.0020013

**Published:** 2006-02-03

**Authors:** Himanshu Sinha, Bradly P Nicholson, Lars M Steinmetz, John H McCusker

**Affiliations:** 1 Department of Molecular Genetics and Microbiology, Duke University Medical Center, Durham, North Carolina, United States of America; 2 European Molecular Biology Laboratory, Heidelberg, Germany; Brandeis University, United States of America

## Abstract

Whether in natural populations or between two unrelated members of a species, most phenotypic variation is quantitative. To analyze such quantitative traits, one must first map the underlying quantitative trait loci. Next, and far more difficult, one must identify the quantitative trait genes (QTGs), characterize QTG interactions, and identify the phenotypically relevant polymorphisms to determine how QTGs contribute to phenotype. In this work, we analyzed three Saccharomyces cerevisiae high-temperature growth (Htg) QTGs *(MKT1, END3,* and *RHO2)*. We observed a high level of genetic interactions among QTGs and strain background. Interestingly, while the *MKT1* and *END3* coding polymorphisms contribute to phenotype, it is the *RHO2* 3′UTR polymorphisms that are phenotypically relevant. Reciprocal hemizygosity analysis of the Htg QTGs in hybrids between S288c and ten unrelated S. cerevisiae strains reveals that the contributions of the Htg QTGs are not conserved in nine other hybrids, which has implications for QTG identification by marker-trait association. Our findings demonstrate the variety and complexity of QTG contributions to phenotype, the impact of genetic background, and the value of quantitative genetic studies in S. cerevisiae.

## Synopsis

Most of the differences in phenotype between unrelated members of a species are polygenic in nature. Because of their ubiquity and importance, these polygenic (or quantitative) traits have been intensively studied, and a variety of techniques have been proposed to identify and characterize quantitative trait genes (QTGs). Indeed, the main application of the recently published human HapMap project is to identify the genes responsible for diseases that are quantitative in nature. Using a well-defined Saccharomyces cerevisiae quantitative trait locus containing three QTGs *(MKT1, END3,* and *RHO2)*, the authors used deletions to analyze the contributions of each gene to phenotype, singly and in combination, and found a variety of interactions. Expression analysis showed no difference in steady-state mRNA levels between alleles of the three genes. Homologous allele replacement identified the phenotypically relevant differences between alleles of each gene, which were single coding polymorphisms for two genes *(MKT1* and *END3)* and the 3′ untranslated region of one gene *(RHO2)*. Finally, analysis of multiple genetic backgrounds showed that the phenotypes conferred by these genetic variants were not conserved. The results show that the techniques proposed to identify QTGs, such as expression analysis and marker-trait association, have profound limitations, and that unbiased genome-wide approaches are needed to dissect quantitative traits. The results also demonstrate the complexity of the genetic interactions that affect quantitative traits and the value of the S. cerevisiae system in studying these traits.

## Introduction

Identification of quantitative trait genes (QTGs) has been hampered by multiple factors, including variable quantitative trait locus (QTL) contributions, complex architectures [1,2], epistasis [3,4], gene-environment interactions, and genetic heterogeneity [5–7]. With the availability of whole genome sequences, several strategies have been proposed to facilitate the identification of QTGs [7–12], including marker-trait association, systematic functional screening, and candidate gene prediction by expression profiling or phenotype screening [13]. However, the major gap in QTL analysis is the lack of detailed molecular information for any one quantitative trait regarding QTL structure, genetic interactions, and the contributions (or relevance) of QTGs identified in one genetic background or population to other genetic backgrounds or populations of the same species.

We previously mapped a Saccharomyces cerevisiae high-temperature growth (Htg) QTL and identified *MKT1, END3,* and *RHO2* as Htg QTGs [1]. The QTL complexity was surprising not only because there were three QTGs in the 32-kb interval, but also because the contributing alleles derived from both the Htg^+^ YJM145 (alleles designated as *MKT1–145* and *RHO2–145*) and Htg^−^ S288c (*END3–288*) backgrounds. In the YJM145, S288c, and 11 other Htg^+^ and Htg^−^ genetic backgrounds, there were multiple coding and noncoding QTG polymorphisms; however, by marker-trait association the phenotypically relevant polymorphisms were not identifiable [1].

**Figure 1:**
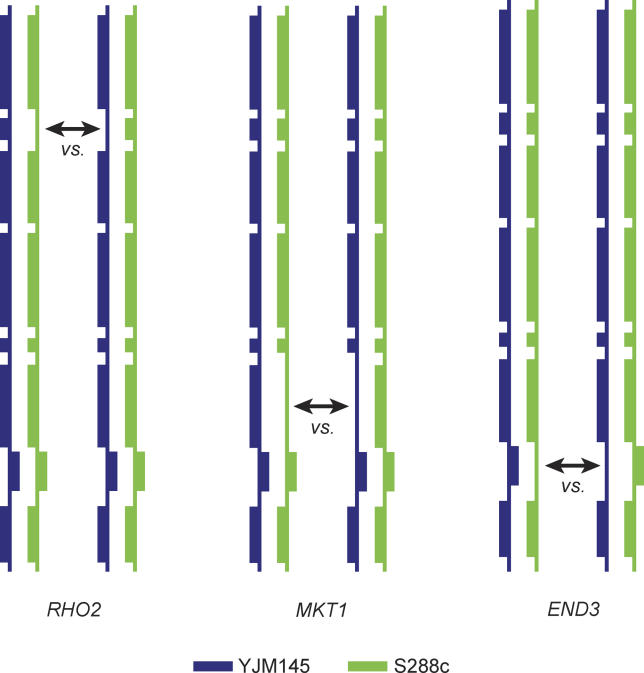
Reciprocal Hemizygosity Analysis The vertical lines represent the chromosome XIV QTL, and the boxes on the line represent individual genes in the Htg QTL. In RHA, for each Htg QTG *(RHO2, MKT1,* and *END3),* one allele of each QTG (or putative QTG) is deleted in a hybrid strain resulting in a pair of deletion heterozygotes (hemizygotes) that have one intact QTG allele from each parent. RHA competitions between the strains in a pair are done to determine the contribution of each Htg allele to the Htg phenotype.

In this study, we assessed genes encoding genetic or physical interactors of *MKT1, RHO2,* and *END3* as novel QTGs and found that none of these genes are Htg QTGs; we also characterized the three QTGs in isolation and in different combinations using deletion and reciprocal hemizygosity analysis (RHA; see Figure 1) [1]. We performed expression and homologous allele replacement analysis to identify phenotypically relevant polymorphisms and used RHA to determine allele contributions to Htg in ten different genetic backgrounds. Our study sheds light on the complexity underlying quantitative traits and emphasizes the utility of different approaches to dissect and analyze quantitative traits.

## Results

### Known Genetic and Physical Interactors of the Htg QTGs Are Not Htg QTGs Themselves

One potential strategy to identify novel QTGs is to test genes that interact genetically (e.g., synthetic lethality) or physically (e.g., in complexes) with known QTGs. No genetic interactions have been reported for *mkt1* or *rho2* mutations [14,15]. However, *end3* has been shown to be synthetically lethal with *apm3* [16], *arp2* [17], and *pan1* [18] mutations and has a synthetic slow-growth phenotype with *rvs161Δ* and *rvs167Δ* deletions [19]. End3p also physically interacts with Pan1p [18] and, in addition, Mkt1p physically interacts with Pbp1p [20]. Therefore, we performed RHA in YJM145/S288c hybrids to determine if these genes, the gene products of which interact genetically or physically with the gene products of known Htg QTGs, are Htg QTGs themselves. However, RHA showed that none of these interacting genes make allele-specific contributions to the Htg phenotype; that is, *APM3, ARP2, PAN1, RVS161, RVS167,* and *PBP1* are not Htg QTGs.

### Deletion Analysis of Htg QTGs

To determine the deletion phenotypes of Htg QTGs in the S288c and YJM145 backgrounds, we constructed single (*qtg1Δ*/*qtg1Δ*) and double (*qtg1Δ*/*qtg1Δ qtg2Δ*/*qtg2Δ*) diploid deletion strains and tested their Htg phenotypes. Consistent with previous work [21,22], *end3*Δ S288c and YJM145 background strains were temperature-sensitive at 37 °C. Therefore, End3p is required for growth at even modestly elevated temperatures in both backgrounds.

In the S288c background at 39 °C, the highest temperature tested in this Htg^−^ background, *rho2Δ* strains grew better than wild-type strains. However, in the Htg^+^ YJM145 background, *rho2Δ* strains grew less than wild type at 40 °C and 41 °C (Table 1). In the S288c background, *mkt1Δ* and wild-type strains grew equivalently at 39 °C. However, in the YJM145 background, *mkt1Δ* strains grew less than wild type at 40 °C, while at 41 °C *mkt1Δ* strains did not grow. Therefore, while *end3*Δ mutants in both parental backgrounds were unable to grow at 37 °C, the *rho2*Δ and *mkt1*Δ phenotypes were different in the two genetic backgrounds, which suggests that phenotype screening of null mutants may have limited predictive value as a QTG identification strategy.

**Table 1:**
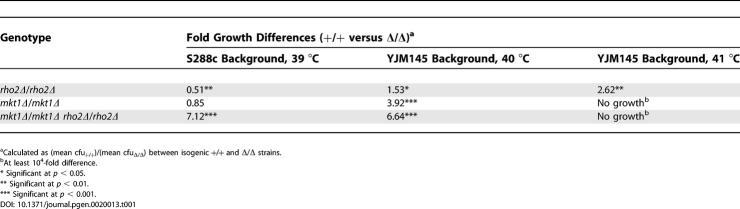
Phenotypes of Htg QTG Deletion Strains in the Parental Backgrounds

In both the S288c and YJM145 backgrounds, we constructed double mutants to test for synthetic interactions between individual Htg QTGs. No synthetic lethal interactions were noted for any pair of deletions or for deletion of all three Htg QTGs in either background. All *end3*Δ-containing strains (*end3*Δ *mkt1*Δ, *end3*Δ *rho2*Δ, and *end3*Δ *mkt1*Δ *rho2*Δ) grew similarly to *end3*Δ strains in both backgrounds. However, in contrast to *rho2*Δ and *mkt1*Δ strains, *mkt1*Δ *rho2*Δ strains in the S288c background grew less than wild type at 39 °C, and thus exhibit a synthetic Htg^−^ phenotype (Table 1). When tested in the YJM145 background at 40 °C, however, the growth defect of *mkt1*Δ *rho2*Δ strains was the sum of the *mkt1*Δ and *rho2*Δ growth defects, consistent with Mkt1-145p and Rho2-145p contributing additively to Htg (Table 1).

### Genetic Interactions between Htg QTG Alleles

As the first step in assessing the genetic interactions between Htg QTG alleles, we determined the Htg phenotypes of a heteroallelic YJM145/S288c hybrid versus deletion heterozygotes; for example, *END3–145*/*END3–288* versus *END3–145*/*Δ*, and *END3–145*/*END3–288* versus *Δ*/*END3–288*. Consistent with the Htg^+^ alleles of *MKT1, END3,* and *RHO2* being dominant, deletion heterozygotes containing only the Htg^+^ QTG alleles had Htg phenotypes similar to a heteroallelic hybrid containing both Htg^+^ and Htg^−^ alleles (Table 2). As predicted from our previous work (Table S1) [1], deletion heterozygotes containing only an Htg^−^ allele had a highly significant growth disadvantage at high temperature relative to a heteroallelic hybrid.

**Table 2:**
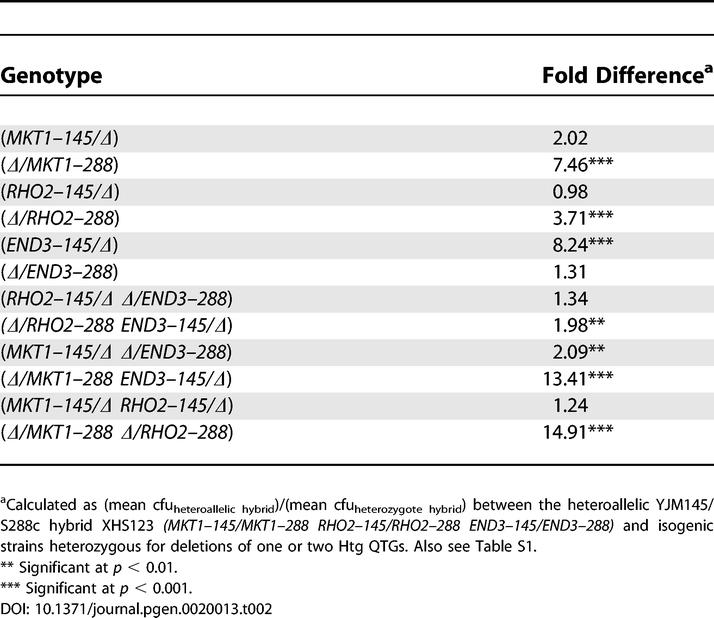
Growth Difference at 41 °C between a Heteroallellic Hybrid and Hybrids Heterozygous for Deletions of One or Two Htg QTGs

Having determined the Htg phenotypes of a heteroallelic hybrid versus all single-deletion heterozygotes, we tested for interactions between Htg QTG alleles by determining the relative Htg phenotypes of a heteroallelic hybrid versus double-deletion heterozygotes. In competitions of a heteroallelic hybrid versus hybrids with the Htg^−^ alleles of *RHO2* and *END3* deleted (*RHO2–145/Δ Δ/END3–288*), there was no difference between the hybrids. In contrast, in competitions of the heteroallelic hybrid versus hybrids with the Htg^+^ alleles of *RHO2* and *END3* deleted (*Δ/RHO2–288 END3–145/Δ*), the deletion heterozygotes were Htg^−^ relative to the heteroallelic hybrid. However, the approximately two-fold growth differential in these competitions was much less than that observed when single Htg^+^ alleles of *RHO2* and *END3* were deleted (Table 2), suggesting a complex genetic interaction between alleles of *RHO2* and *END3*.

In competitions of a heteroallelic hybrid versus hybrids with Htg^+^ alleles of *MKT1* and *END3* deleted (*Δ/MKT1–288 END3–145/Δ*), the deletion heterozygotes had Htg phenotypes significantly less than the heteroallelic hybrid. The Htg phenotypic differential in these competitions was approximately equal to the sum of the differences observed in the single-deletion heterozygote analyses (Table 2), indicating *MKT1–145* and *END3–288* additivity. Similarly, in competitions of a heteroallelic hybrid versus hybrids with Htg^+^ alleles of *MKT1* and *RHO2* deleted (*Δ/MKT1–288 Δ/RHO2–288*), the deletion heterozygotes had Htg phenotypes significantly less than the heteroallelic hybrid, consistent with the earlier observed *MKT1–145* and *RHO2–145* additivity.

### Expression Analysis of the Htg QTGs

To further characterize the Htg QTGs and gain insight into the mechanism of the quantitative trait and the location(s) of the phenotypically relevant polymorphism(s), we carefully examined the steady state mRNA levels of the three Htg QTGs as a function of both temperature and allele. Since isogenic reciprocal hemizygotes contain only one allele of a QTG, in a comparison between reciprocally hemizygous strains, any allele-specific differences in steady state mRNA levels must be due to *cis*-acting polymorphisms. Therefore, we performed Northern analysis of two pairs of reciprocal hemizygotes for each QTG. Temperature (25 °C versus 37 °C) had no effect on *RHO2* and *END3* steady state mRNA levels. However, *MKT1* steady state mRNA levels increased in an allele-nonspecific manner at 37 °C (37 °C/25 °C = 2.0). No allele-specific fold differences larger than two-fold were observed in steady state mRNA at either temperature for any of the genes.

### Homologous Allele Replacement of the Htg QTGs

As a first test of the hypothesis that the phenotypically relevant differences between Htg^+^ and Htg^−^ QTG alleles were contained within the genes, and most likely from expression results within the coding regions, we ectopically integrated alleles of *RHO2* and alleles of *MKT1* (separately) at the *HO* locus. However, we found that the resulting strains had insert-specific, Htg QTG allele-nonspecific, dominant-negative growth defects (unpublished data).

Since these ectopic integrations were problematic, we then constructed isogenic homologous allele replacement strains that differed (see Table S2) solely with respect to the S288c versus YJM145 nonsynonymous coding region polymorphisms and one 5′ and one 3′ UTR polymorphism in *MKT1* (a−104g D30G K453R a+54t), or nonsynonymous coding polymorphisms in *END3* (S258N D268N) or *RHO2* (F91C) (see Table S3). We then compared the Htg phenotypes of isogenic strains that differed solely in these *MKT1, END3,* or *RHO2* polymorphisms.

For isogenic S288c, YJM145 and YJM145/S288c hybrid background strains with different *RHO2* alleles, there were no statistically significant differences in Htg phenotype (Table 3). Therefore, the single nonsynonymous *RHO2* coding polymorphism (C91F) is phenotypically neutral. Since there are no *RHO2* 5′UTR polymorphisms, this suggested that *RHO2* 3′UTR polymorphisms contribute to phenotype (see Table S3 for polymorphisms).

**Table 3:**
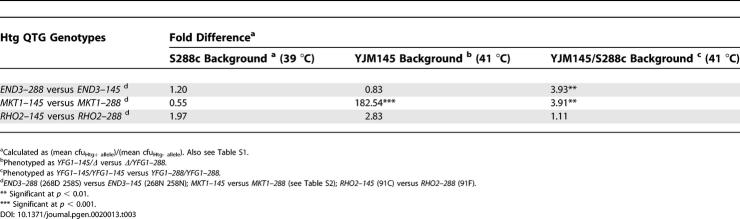
Growth Difference between Coding Allele Exchange Strains for Htg Genes in the S288c, YJM145, and YJM145/S288c Hybrid Backgrounds

To test the *RHO2* 3′UTR hypothesis, we replaced the 3′UTR of *RHO2–288* with the 3′UTR of *RHO2–145*; that is, we constructed *RHO2–288* 3′UTR-145 strains. We then performed Htg competitions between heteroallelic hybrids (*RHO2–145* 3′UTR-145/*RHO2–288* 3′UTR-288) versus hybrids with the S288c-derived *RHO2* 3′UTR (Δ/*RHO2–288* 3′UTR-288) versus hybrids with the YJM145-derived *RHO2* 3′UTR (*Δ*/*RHO2–288* 3′UTR-145). Consistent with our RHA results, the *Δ*/*RHO2–288* 3′UTR-145 strains grew as well as the heteroallelic hybrids, while the growth difference between Δ/*RHO2–288* 3′UTR-288 strains and the heteroallelic hybrid was similar to the growth difference between Δ/*RHO2–288* 3′UTR-288 and *Δ*/*RHO2–288* 3′UTR-145 strains (Table 4). Therefore, the *RHO2* 3′UTR is primarily and possibly solely responsible for the allele-specific contribution of *RHO2* to the Htg phenotype.

**Table 4:**
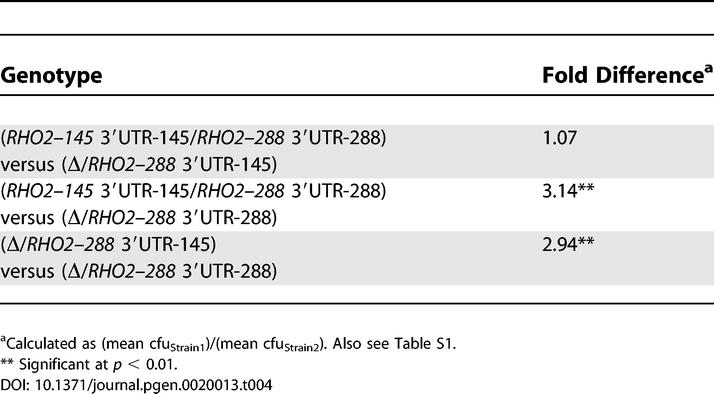
Growth Difference at 41 °C between YJM145/S288c Hybrids Containing YJM145- versus S288c-Derived *RHO2* 3′UTRs

For both isogenic S288c and YJM145 background strains with different *END3* coding alleles, there were no significant differences in Htg phenotype (Table 3). However, consistent with our previous results [1], Htg^+^ *END3–288* allele-containing YJM145/S288c hybrid background strains had highly significant Htg advantages over Htg^−^ *END3–145* allele-containing strains.

In the S288c background, there was no significant difference in growth between strains with different *MKT1* alleles (Tables 3 and S2). However, in the YJM145 background, Htg^+^ *MKT1–145* allele-containing strains had highly significant Htg advantages over Htg^−^ *MKT1–288* allele-containing strains. Similarly, in the YJM145/S288c hybrid background, Htg^+^ *MKT1–145* allele-containing strains had highly significant Htg advantages over Htg^−^ *MKT1–288* allele-containing strains. Therefore, these *END3* coding (S258N D268N) and *MKT1* coding/noncoding (a−104g D30G K453R a+54t) polymorphisms are responsible for much of the *END3* and *MKT1* allele-specific contributions to the Htg phenotype, and their effects are highly influenced by genetic background.

### Identification of the *MKT1* and *END3* Quantitative Trait Nucleotide

To determine which of the coding single-nucleotide polymorphisms (SNPs) in the two major Htg QTGs, *MKT1* and *END3,* are relevant quantitative trait nucleotides (QTNs) for the Htg phenotype, we used site-directed mutagenesis to replace the two coding SNPs in *MKT1* and *END3* individually. The isogenic strains were created such that they differed solely in the coding SNP under investigation. RHA competitions were done to determine the effect of each SNP replacement in each gene on the Htg phenotype.

For *MKT1,* RHA analysis of SNP replacement strains showed that the A1358G SNP (where the S288c base-nucleotide numbers from the start-YJM145 base), resulting in a conservative amino acid change from lysine (K) in S288c to arginine (R) in YJM145 (K453R), had no effect on Htg. However, the A89G SNP, resulting in a nonconservative amino acid change from aspartic acid (D) in S288c to glycine (G) in YJM145 (D30G), affected Htg with 89G (30G)-containing strains growing better at high temperature (Table 5).

**Table 5:**
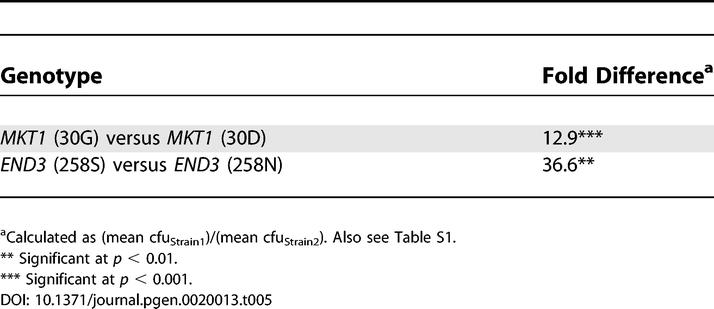
Growth Difference at 41 °C between YJM145/S288c Hybrids Containing YJM145- versus S288c-Derived *MKT1* and *END3* Coding Polymorphisms

For *END3,* RHA analysis of SNP replacement strains showed that the C802T SNP, resulting in an amino acid change from aspartic acid (D) in S288c to asparagine (N) in YJM145 (D268N), had no effect on Htg. However, the C773T SNP, resulting in a nonconservative amino acid change from serine (S) in S288c to asparagine (N) in YJM145 (S258N), affected Htg with 773C (258S)-containing strains growing better at high temperature (Table 5).

### Phenotypic Heterogeneity among the Htg QTGs in Diverse Genetic Backgrounds

Marker-trait association studies assess the association between a sequence variant and a trait. Marker-trait association, which may be affected by genetic background [2,3] and is one of the applications of the data coming from the HapMap project, aims to aid QTG discovery [23]. As a first step in determining the impact of genetic background on phenotype and on marker-trait association, we determined the Htg phenotype of the S288c background and of ten additional unrelated genetic backgrounds. The Htg phenotypes of the 11 unrelated genetic background strains varied over a greater than 10^4^-fold range, demonstrating a high degree of Htg phenotypic heterogeneity, and there was no association between the specific *MKT1, RHO2,* and *END3* polymorphisms shown to be relevant in YJM145/S288c and the Htg phenotype (Table 6).

**Table 6:**
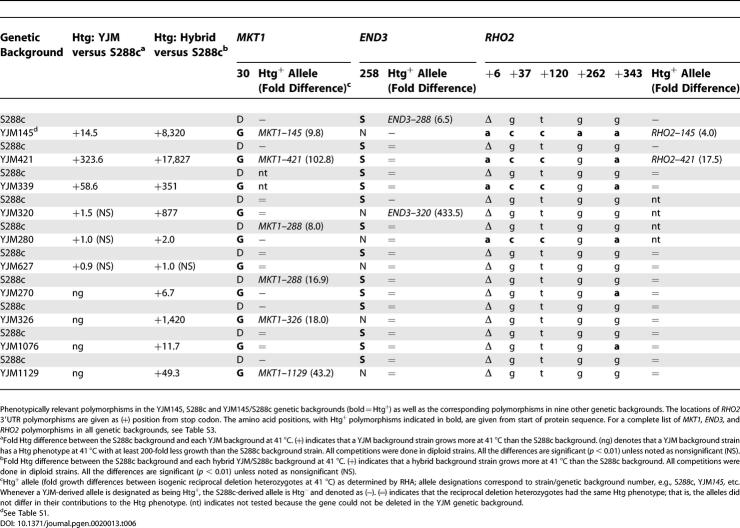
Htg Phenotypes and RHA of Htg Genes in Hybrids between S288c and Other S. cerevisiae Genetic Backgrounds

To further assess the impact of genetic background on phenotype and on marker-trait association, we crossed an S288c background strain with strains from each of the ten unrelated Htg^+^ and Htg^−^ genetic backgrounds and determined the Htg phenotypes of the resulting hybrids. All hybrids, except YJM627/S288c, showed positive heterosis (Table 6); that is, the Htg phenotype of the hybrids was greater than either parent, consistent with a general benefit of heterozygosity at many loci. Therefore, although the S288c genome constitutes one-half of the genome content of all hybrids, the genetic composition of the entire genome affects the Htg phenotype. Again, there was no association between the specific *MKT1, RHO2,* and *END3* polymorphisms shown to be relevant in YJM145/S288c and Htg phenotype.

As a final step in determining the impact of genetic background on phenotype and on marker-trait association, we performed RHA of the Htg QTGs in the ten hybrid genetic backgrounds (Table 6) [1]. RHA of *MKT1, END3,* and *RHO2* in hybrids between S288c and the ten unrelated genetic backgrounds further demonstrated the complex impact of genetic background on QTG contributions to phenotype. For example, in *MKT1,* all of the non-S288c backgrounds have the same coding polymorphism (30G) that was found to be Htg^+^ in the YJM145/S288c hybrid. However, in the hybrids between S288c (30D) and these other genetic backgrounds, the 30G-containing *MKT1* alleles present in all of the hybrids could be phenotypically Htg^+^, neutral, or Htg^−^ and, conversely, the *MKT1–288* (30D) allele present in all of the hybrids could be Htg^−^, neutral, or Htg^+^. There are similar cases for the *END3* Htg^+^ coding polymorphism 258S. Our analysis shows therefore that QTGs identified in one background do not necessarily make the same contribution to phenotype in other genetic backgrounds, and that QTL architecture (that is, the identity and arrangement of QTGs) is therefore not conserved and instead varies with genetic background.

## Discussion

Here we greatly extended our previous analysis [1] by determining the genetic interactions between the Htg QTGs and identifying the QTNs in the Htg genes. We determined the conservation of these QTNs in various strain backgrounds and tested various methods of QTG identification and analysis.

Our results show that not only is the architecture of this QTL complex, but that genetic interactions further add to the complexity. We found both additive interactions, such as between *MKT1* and *RHO2,* and more complex genetic interactions, between *END3* and *RHO2*. In addition, we showed that alleles have different effects in different genetic backgrounds. For example, *END3–288* and *END3–145* are phenotypically equivalent in the parental backgrounds, but differ phenotypically in the hybrid background. That is, alleles of genes from both parental backgrounds are needed to produce the *END3–288* versus *END3–145* phenotypic differential.

We find that none of the known interacting genes of the Htg QTGs are themselves Htg QTGs. We also find that none of the three Htg QTGs differ in their steady state mRNA levels. Indeed, our results show that *RHO2* 3′UTR polymorphisms that have no effect on steady state mRNA levels but may instead affect mRNA translation [24,25] or localization [26] contribute to Htg; as with QTGs containing coding QTNs, such QTGs are not identifiable by expression analysis. Since the above functional and expression techniques form the basis for candidate gene approaches to identify QTGs, our results demonstrate the need for unbiased genome-wide approaches to identify QTGs.

These three Htg QTGs have no obvious functional relationship, which may have implications for other quantitative traits. *END3,* which has a temperature-sensitive deletion phenotype [22] and a phenotypically relevant coding polymorphism, is involved in endocytosis. *RHO2* encodes a nonessential small GTPase [27]; we found that no allele-specific differences exist in *RHO2* steady state mRNA levels and that the *RHO2* 3′UTR is phenotypically relevant. *MKT1* is involved in the maintenance of the M2 dsRNA satellite of the L-A virus; the S288c-allele is thought to be a loss-of-function mutation [28]. *MKT1* also plays a role in the posttranscriptional regulation of *HO* by binding to the 3′UTR of *HO* mRNA, without affecting steady state mRNA levels [20]. We find that the *MKT1* D30G polymorphism affects Htg and others have shown that this same *MKT1* polymorphism also plays a role in sporulation efficiency [29]. Since none of the *mkt1* mutant phenotypes appear to be related, this represents an intriguing case of pleiotropy. This *mkt1* mutant pleiotropy may be related to the function of Mkt1p in binding to the 3′UTR of *HO* mRNA and controlling translation (without affecting steady state mRNA levels): in addition to *HO,* Mkt1p may regulate the translation of other mRNAs, such as *RHO2*.

Our analysis of Htg allele contributions in multiple genetic backgrounds demonstrates the impact of genetic background on allelic phenotype. Although our sample size was small, the lack of association of the sequence variants to the contribution of an allele has implications for marker-trait association. A variety of theoretical and experimental studies of QTLs in higher organisms [5,7,8,30] have suggested that epistasis and/or genetic background is a complicating factor in QTG identification; our results provide a graphic demonstration of these effects. To fully understand a phenotype, one must understand the background effects on phenotype, which requires the identification of the genetic variants in each background that are causative. One way to identify the causative genetic variants is to “homozygose” known QTGs, and then map novel QTLs; another is to backcross F1 segregants and map novel Htg QTLs. These strategies will assist in identifying additional Htg QTLs that contribute to phenotype in the presence and absence of the identified Htg QTGs.

Our results show the importance of yeast as a model system for QTL analysis and highlight important issues related to QTL identification and analysis. Our results and conclusions from quantitative studies in yeast show that the study of these phenotypes in higher organisms remains a difficult challenge. Improved understanding of quantitative traits in simpler eukaryotes such as yeast will facilitate the analysis of complex traits in higher eukaryotes.

## Materials and Methods

### Yeast strains, growth media, and conditions.

Yeast strains were grown at 30 °C unless otherwise stated. The S. cerevisiae strains used in this work (Table S4) are derivatives of S288c (a laboratory genetic background, designated “S”), YJM145 (a clinically-derived genetic background, designated “YBN,” “YJM,” or “YHS” [31]), and other clinically and nonclinically derived genetic backgrounds (designated YHS, YRP, and YJM). To make hybrids (designated as “XHS”) of test and S288c background strains, spores of *lys2* and *lys5* diploid strains were allowed to mate on YPD plates that were then replica plated to SDC-LYS plates to select Lys^+^ diploid hybrids.

YPD (yeast extract peptone and dextrose), YPEG (yeast extract peptone ethanol-glycerol), SD (synthetic dextrose minimal), SDC (synthetic dextrose complete) [32], and α-aminoadipate media [33] have been described previously. Filter-sterilized antibiotics (hygromycin 300 μg/ml, nourseothricin 100 μg/ml, G418 200 μg/ml) were added to YPD after autoclaving [34,35]. Methomyl (Chem Service, West Chester, Pennsylvania, United States) was added to YPEG medium (1 mM final concentration) that had cooled to about 50 °C; plates were poured immediately after mixing.

To induce sporulation, cultures were first grown overnight in liquid YPD at 30 °C, after which a 1-ml aliquot was removed, washed in sterile water twice, and resuspended in 3 ml of sterile 0.5% (w/v) potassium acetate medium [36]. Sporulating cultures were incubated at 30 °C for 48 h with aeration in a roller drum and examined microscopically for the presence of tetrads.

High-temperature growth competitions between two or three strains were performed using a technique similar to RHA, as previously described [1].

### PCR and yeast transformation.

The PCR to make gene deletion constructs containing dominant drug-resistance markers was done as described previously [35]. All PCR products were purified with Qiagen's PCR Purification kit (Qiagen, Valencia, California, United States) before introduction into yeast. DNA was introduced into yeast by the lithium acetate transformation protocol [37]. Homologous integration of dominant drug-resistance cassettes was verified by colony PCR [38]. In cases where colony PCR did not yield a product, genomic DNA was isolated [39] and PCR was performed using colony PCR conditions. The primers used in the study (IDT, Corwallis, Illinois, United States) are listed in Table S5.

### Reciprocal hemizygosity analysis.

RHA, a technique for identifying allele-specific contributions to a phenotype, was performed as previously described (see Figure 1) [1]. The resulting data were used to calculate means and standard errors and to perform tests of significance [40].

### Reciprocal hemizygosity expression analysis.

To assay the expression levels of each allele of the Htg quantitative trait genes, we performed Northern analysis of isogenic reciprocal hemizygotes. For each Htg quantitative trait gene, a pair of reciprocal hemizygotes were grown (separately) at 25 °C and 37 °C in 50 ml of YPD with aeration to an OD_600_ of 1.0. Total RNA was extracted as previously described, with the following modifications. After hot phenol-chloroform extraction, samples were frozen overnight at −80 °C and then centrifuged for 15 min at 13,000 rpm; the aqueous phase was processed as previously described. Cells were then washed and resuspended in 400 μl of extraction buffer (50 mM sodium acetate [pH 5.3], 1 mM EDTA) and 40 μl of SDS (10% w/v) followed by extraction with 440 μl of hot buffered phenol-chloroform (65 °C) for 30 min with vortexing every 5 min. After being frozen overnight at −80 °C, samples were centrifuged for 15 min at 13,000 rpm. After transfer of the aqueous phase into a new tube, the samples were phenol-chloroform extracted once. The RNA was precipitated with two volumes of 100% ethanol and 0.1 volume of 3 M sodium acetate (pH 5.3) for 30 min at −20 °C. After centrifugation, the RNA was resuspended in 100 μl of DEPC-treated water. For each sample, 10 μg of RNA was size-fractionated on a MOPS/formaldehyde gel and Northern blotting was performed. The membranes were probed with ^32^P-labeled gene-specific PCR probe in ULTRAhyb solution (Ambion, Austin, Texas, United States) and were incubated overnight at 42 °C. The blots were washed twice in 2× SSC and 0.1% SDS for 5 min and twice again with 0.1× SSC and 0.1% SDS for 15 min, exposed to a phosphor-imager screen that was read with a Storm imaging system and quantified using ImageQuant software (Amersham Biosciences, Piscataway, New Jersey, United States). The mRNA levels of the Htg quantitative trait genes were compared against actin mRNA as a loading control.

### Cloning alleles of quantitative trait genes.

The Htg^+^ and Htg^−^ alleles of *MKT1-END3* (pHS07 and pHS08; see Figure S1) and *RHO2* (pHS09 and pHS10; see Figure S2) were cloned using a modified gap repair [41] technique. To gap repair cloned alleles of each gene, primers with 5′ tails homologous to the pRS316 [42] multicloning site were used to amplify from genomic DNA 0.5-kb regions of the open reading frames of the genes upstream and downstream of the Htg QTG. The PCR products were then purified using a Qiagen PCR Purification kit and introduced into *ura3* strains (S19 and YJM791, Table S4) along with SmaI-digested pRS316 plasmid. Circularization of the SmaI-digested plasmid occurred via in vivo recombination, with the upstream and downstream PCR products providing homology between the multicloning site and the chromosomal regions flanking the Htg gene. Plasmids from Ura^+^ transformants, which were selected on SDC-URA plates, were introduced into the E. coli strain DH5α followed by selection on LB (containing 100 μg/ml ampicillin and 40 μg/ml X-gal) [43,44]. Plasmids were isolated from white, ampicillin-resistant E. coli transformants using the Qiagen Miniprep kit and were checked by restriction digestion for the presence of the desired inserts. The inserts were then sequenced to confirm their sequence and the presence of the allele-specific polymorphisms.

### Homologous coding allele replacements of Htg QTGs.

Homologous allele replacements were done in haploid strains in three different ways for the three Htg genes. For *MKT1* coding and noncoding allele replacements, a *LYS5* counterselection method described by Ito-Harashima and McCusker [45] was used. A region of the *MKT1* gene from 69 to 1,379 bp was first deleted in haploid *lys5* strains (YHS731 and S1288) using the *LYS5*MX4 cassette to generate *mkt1Δ*::*LYS5*MX4 strains. Into these *mkt1Δ*::*LYS5*MX4 strains were introduced (i) a 3,344-bp BcgI-BsrGI *MKT1* allele fragment from either pHS07 (*CEN URA3 MKT1–288 END3–288*) or pHS08 (*CEN URA3 MKT1–145 END3–145*) (see Figure S1) and (ii) pSA41 (*CEN* NatMX4) (see Figure S3); cells were plated on YPD+Nat plates. Replacements of the *mkt1Δ*::*LYS5*MX4 by the cloned *MKT1* DNA were selected by replica plating the colonies growing on YPD+Nat onto α-aminoadipate plates; replica plating to α-aminoadipate plates was repeated to reduce background growth. Lys^−^ colonies growing on α-aminoadipate plates were tested by PCR for the desired allele replacement, e.g., *mkt1Δ*::*LYS5*MX4 *lys5* → *MKT1–145 lys5*. DNA sequencing was performed to confirm the presence of the desired allelic polymorphism (see Table S2).

*RHO2* and *MKT1* coding allele replacements were performed in the manner similar to the *MKT1* coding and noncoding allele replacements except that a Hyg/T-*urf13*MX4 cassette (in pBN49, see Figure S4) was used instead of *LYS5*MX4 for selection and counterselection. The Hyg/T-*urf13*MX4 cassette is a counterselectable drug marker cassette in which hygromycin resistance is used to select transformants, after which the cassette can be counterselected on methomyl-containing medium; T-*urf13* is a maize mitochondrial gene that confers sensitivity to methomyl [46]; a similar pop-in/pop-out gene replacement strategy has been described in Pichia pastoris [47]. For *MKT1,* the polymorphic regions of 69–104 bp, containing the coding polymorphism at 89 bp, and 1,339–1,379, containing the coding polymorphism at 1,358 bp, were deleted separately using the Hyg/T-*urf13*MX4 cassette. For *RHO2,* the polymorphic region of 216–324 bp containing the coding polymorphism at 271 bp was deleted in haploid strains using the Hyg/T-*urf13*MX4 cassette. Using a technique similar to that described by Storici et al. [48], we introduced a 120-bp dsDNA containing the appropriate allele-specific polymorphism into these hygromycin-resistant methomyl-sensitive *rho2*Δ::Hyg/T-*urf13*MX4 transformants. We then selected for loss of Hyg/T-*urf13*MX4 cassette on YPEG + 1 mM methomyl plates. The putative allele replacements were checked for hygromycin sensitivity and by PCR. DNA sequencing was performed to confirm the presence of the desired polymorphisms.

*END3* allele replacements were based on rescuing the temperature-sensitive phenotype of *end3Δ* strains. First, the polymorphic region of *END3* (773–802 bp) was deleted using the KanMX4 cassette to generate *end3Δ*::KanMX4 strains. Into the *end3Δ*::KanMX4 strains was introduced (i) a 120-bp dsDNA containing allele-specific polymorphisms using a technique similar to that described by Storici et al. [48] and (ii) pAG26 [35] (*CEN* HygMX4); cells were plated onto YPD+Hyg and incubated at 30 °C. The hygromycin-resistant transformants were then replica plated onto YPD plates, which were incubated at 39 °C to select for *END3* ^+^ transformants—that is, transformants in which *end3Δ*::KanMX4 has been replaced by the dsDNA repairing the deletion and generating *END3* ^+^. The putative *END3* ^+^ transformants were checked for Kan sensitivity and temperature resistance, and were confirmed by PCR and DNA sequencing.

### Homologous allele replacements of the *RHO2* 3′UTR.

*RHO2* 3′UTR allele replacements were performed by first deleting the *RHO2* 3′UTR, spanning 2 bp upstream of the *RHO2* stop codon to 208 bp upstream of the *TOP2* start codon in strain S795, with a *FCY1*-NatMX4 cassette (in pBN33, see Figure S5), selecting for Nat resistance. The *RHO2* 3′utrΔ::*FCY1*-NatMX4 genotype in strain S3207 was confirmed by PCR. Into the *RHO2* 3′utrΔ::*FCY1*-NatMX4 strain were introduced (i) a 1,172-bp SfiI-BglII, *RHO2* 3′UTR allele fragment from pHS09 and (ii) pBN98 (*CEN* KanMX4) (see Figure S6); cells were plated on YPD+G418 plates. Replacements of the *RHO2* 3′utrΔ::*FCY1*-NatMX4 in S3207 by the cloned *RHO2* 3′UTR DNA were selected by replica plating the colonies growing on YPD+G418 onto SDC plates containing 1 mM 5-fluorocytosine. Colonies resistant to 5-fluorocytosine were confirmed by PCR for the desired allele replacement (i.e., *RHO2–288* 3′utrΔ::*FCY1*-NatMX4 → *RHO2–288* 3′UTR-145), resulting in strains S3209 and S3210.

## Supporting Information

Figure S1 Restriction Map of Plasmids pHS07/pHS08 Showing the Cloned Fragment and Restriction Enzymes Used to Obtain *MKT1* Fragments pHS07 is *CEN URA3 MKT1–288 END3–288* and pHS08 is *CEN URA3 MKT1–145 END3–145* (see Materials and Methods and Table S2). *MKT1* coding polymorphisms are shown at 30 and 453 amino acid residues.
(1.8 MB PSD)

Figure S2 Restriction Map of Plasmids pHS09/pHS10 Showing the Cloned Fragment and Restriction Enzymes Used to Obtain *RHO2* 3′UTR Fragments pHS09 is *CEN URA3 RHO2–145* and pHS10 is *CEN URA3 RHO2–288* (see Materials and Methods and Table S2).
(1.5 MB PSD)

Figure S3 Map of Plasmid pSA41 with *CEN* and NatMX4 Cassette pSA41 is a pAG36-based [35] plasmid.
(1.1 MB PSD)

Figure S4 Restriction Map of Plasmid pBN49 pBN49 is a pFA6-based [34] plasmid containing a novel HygMX4/T-*urf13* cassette. Primers JM41 and JM42 were used to amplify the cassette.
(2.0 MB PSD)

Figure S5 Restriction Map of Plasmid pBN33 pBN33 is a pAG26-based [35] plasmid containing a novel *FCY1*-NatMX4 cassette. Primers JM41 and JM42 were used to amplify the cassette.
(1.8 MB PSD)

Figure S6 Map of Plasmid pBN98 pBN98 is a pXP45-based [49] plasmid with *CEN* and KanMX4 cassettes and a BsrG1 site engineered upstream of Kan start codon.
(348 KB TIF)

Table S1 *MKT1, END3,* and *RHO2* RHA Data (67 KB DOC)

Table S2 *MKT1* Coding/Noncoding Polymorphism Transfers (40 KB DOC)

Table S3 Coding, 5′UTR, and 3′UTR Polymorphisms of *MKT1, END3,* and *RHO2* Alleles in the S288c and YJM Backgrounds (239 KB DOC)

Table S4 List of Strains Used in This Study (623 KB DOC)

Table S5 List of Oligonucleotides and PCR Primers Used in This Study (140 KB DOC)

### Accession Numbers

European Saccharomyces Cerevisiae Archive for Functional Analysis (EUROSCARF; http://web.uni-frankfurt.de/fb15/mikro/euroscarf) accession numbers for the plasmids discussed in this paper are: pSA41 (P30441), pBN33 (P30442), pBN49 (P30443), and pBN98 (P30444).

## Abbreviations

Htg: high-temperature growth
QTG: quantitative trait gene
QTL: quantitative trait locus
QTN: quantitative trait nucleotide
RHA: reciprocal hemizygosity analysis
SNP: single-nucleotide polymorphism
UTR: untranslated region
