# Supplementary material for: Complex Genetic Interactions in a Quantitative Trait Locus
Source: PLoS Genet. 2006 Feb 3;2(2):e13. doi: 10.1371/journal.pgen.0020013 (PMC1359075; doi:10.1371/journal.pgen.0020013)
Supplement: Supplementary file 1 [file pgen.0020013.st001.doc]

**Table S1:** *MKT1*, *END3* and *RHO2* RHA data.

The differences seen in this work between euploid hybrids and hemizygotes with the Htg^+^ alleles of *MKT1*, *RHO2* and *END3* deleted (Table 2) were less than expected from our previous work [1]; differences are also seen between the data in Tables 2, 3, 4 and 5 in this work. Therefore, we retested by RHA all the heterozygous deletion strains of *MKT1*, *RHO2* and *END3* that were used by Steinmetz *et al.* [1]. The results below show that the current difference between the heterozygous deletion pairs of each gene is reduced compared to differences we reported previously [1]. Since the same person (HS) performed all of the RHA in both this and the previous work [1], we believe that a change in the environment (*e.g.* the water or some unknown difference in the manufacturing of one or more media components, the paper on which cells are plated or the Petri dishes used to make plates) is responsible for the differences in phenotype.

Note that the growth differentials for homologous *MKT1* and *END3* coding allele transfers (Table 3) are approximately 40% (*MKT1*) and 60% (*END3*) of the RHA growth differentials shown below; therefore, other polymorphisms may contribute to phenotype. Note that the growth differentials for homologous *RHO2* 3’UTR allele transfers (Table 4) are approximately 80% and 74% of the *MKT1* and *END3* RHA growth differentials shown below. Since there are no other *RHO2* polymorphisms, the single coding polymorphism may make a minor contribution to phenotype.

Fold growth differences, calculated as (mean cfu_Htg+ allele_)/(mean cfu_Htg- allele_), were determined at 41ºC between pairs of YJM145/S288c reciprocally heterozygous deletion hybrid strains.

| Htg QTG genotypes | **Fold difference in Htg phenotype** |
| --- | --- |
| (*MKT1-145*/*∆*) *vs.* (∆/*MKT1-288*) | 9.84*** |
| (*RHO2-145*/*∆*) *vs.* (∆/*RHO2-288*) | 3.96*** |
| (∆/*END3-288*) *vs.* (*END3-145*/*∆*) | 6.46*** |

*** significant at p<0.001

**REFERENCES:**

1. Steinmetz LM, Sinha H, Richards DR, Spiegelman JI, Oefner PJ, et al. (2002) Dissecting the architecture of a quantitative trait locus in yeast. Nature 416: 326-330.
