# Supplementary material for: Complex Genetic Interactions in a Quantitative Trait Locus
Source: PLoS Genet. 2006 Feb 3;2(2):e13. doi: 10.1371/journal.pgen.0020013 (PMC1359075; doi:10.1371/journal.pgen.0020013)
Supplement: Supplementary file 2 [file pgen.0020013.st002.doc]

**Table S2:** *MKT1* coding/non-coding polymorphism transfers.

The transfers of *RHO2* polymorphisms (the separate transfers of the single coding polymorphism and the 3’UTR polymorphisms) and *END3* polymorphisms (two coding polymorphisms) were performed in such a way that only the desired polymorphisms were transferred; that is, for *RHO2*, only the single coding polymorphism or only the 3’UTR polymorphisms were transferred, and for *END3*, only the two coding polymorphisms were transferred. However, for transfers of *MKT1* coding polymorphisms, it was also possible to transfer the single 5’UTR polymorphism or either of the 3’UTR polymorphisms. The parental and allele transfer 5’ and 3’UTR *MKT1* sequences are shown below:

5’UTR (-104)	3’UTR (+54)	3’UTR (+334)

S288c *MKT1-288* UTR	A	A	A

YJM145 *MKT1-145* UTR	G	T	∆

# S288c background *MKT1-145* allele transfer strains

S1652	G	T	A

S1653	A	T	A

# YJM145 background *MKT1-288* allele transfer strains

YHS806	A	A	∆

YHS807	A	A	∆

The 3’UTR (+334) polymorphism, which lies beyond the predicted *MKT1* terminator region, was not transferred in any of the allele transfer strains. Since the 3’UTR (+54) was transferred with the two coding polymorphisms in all of the *MKT1* allele transfer strains, the 3’UTR (+54) polymorphism may contribute to phenotype. The 5’UTR (-104) polymorphism was transferred in three of the four *MKT1* coding polymorphism transfers. In the S288c background strains containing *MKT1-145* with different 5’UTR polymorphisms, there was no difference in phenotype between the allele transfers.
