# Supplementary material for: Complex Genetic Interactions in a Quantitative Trait Locus
Source: PLoS Genet. 2006 Feb 3;2(2):e13. doi: 10.1371/journal.pgen.0020013 (PMC1359075; doi:10.1371/journal.pgen.0020013)
Supplement: Supplementary file 3 [file pgen.0020013.st003.doc]

**Table S3:** Coding, 5’UTR and 3’UTR polymorphisms of *MKT1*, *END3* and *RHO2* alleles the S288c and YJM backgrounds.

Sequence data is derived from Steinmetz *et al.* [1]. Coding polymorphisms are in bold capital letters with coding positions indicated in “AA position” column. All UTR polymorphisms are shown as lower case nucleotide and is defined as intergenic region up to the adjacent ORFs. The locations of 5’UTR and 3’UTR polymorphisms are given as (i) (-) position from ATG start codon and (ii) (+) position from stop codon, respectively.

| ORF | NT position | S288c | YJM145 | YJM270 | YJM280 | YJM320 | YJM326 | YJM339 | YJM421 | YJM627 | YJM1076 | YJM1129 | AA position | S288c codon | Other codon |
| --- | --- | --- | --- | --- | --- | --- | --- | --- | --- | --- | --- | --- | --- | --- | --- |
|  | -363 | a | a | a | g | a | a | a | a | a | a | a |  |  |  |
|  | -272 | t | t | c | t | t | t | t | t | t | c | c |  |  |  |
|  | -231 | a | a | a | a | a | a | a | t | a | a | a |  |  |  |
|  | -215 | c | c | c | c | c | c | c | t | c | c | c |  |  |  |
|  | -212 | t | t | t | t | t | t | t | t | t | t | c |  |  |  |
|  | -194 | t | t | t | t | a | a | t | t | t | t | t |  |  |  |
|  | -154 | a | a | a | a | a | a | a | g | a | a | a |  |  |  |
|  | -130 | a | a | g | a | a | a | a | a | g | g | g |  |  |  |
|  | -35 | g | g | g | g | g | g | g | a | g | g | g |  |  |  |
| ***RHO2*** | 271 | **T** | **G** | **T** | **T** | **T** | **T** | **T** | **T** | **T** | **T** | **T** | 91 | F | C |
| ***RHO2*** | 311 | **G** | **A** | **G** | **G** | **G** | **G** | **A** | **A** | **G** | **G** | **G** | 104 | E | E |
| ***RHO2*** | 428 | **A** | **G** | **A** | **G** | **A** | **A** | **G** | **G** | **A** | **A** | **A** | 143 | A | A |
| ***RHO2*** | 447 | **A** | **A** | **G** | **A** | **A** | **A** | **A** | **A** | **A** | **A** | **A** | 150 | I | V |
| ***RHO2*** | 527 | **C** | **C** | **C** | **C** | **C** | **C** | **C** | **C** | **C** | **T** | **C** | 176 | T | T |
|  | +6 | ∆ | a | ∆ | a | ∆ | ∆ | a | a | ∆ | ∆ | ∆ |  |  |  |
|  | +37 | g | c | g | c | g | g | c | c | g | g | g |  |  |  |
|  | +99 | a | a | g | a | a | a | a | a | a | a | a |  |  |  |
|  | +119 | t | c | t | c | t | t | c | c | t | t | t |  |  |  |
|  | +261 | g | a | g | g | g | g | g | g | g | g | g |  |  |  |
|  | +341 | g | a | a | a | g | g | a | a | g | a | g |  |  |  |
|  | | | | | | | | | | | | | | | |
|  | -386 | c | c | c | c | c | c | c | c | c | g | c |  |  |  |
|  | -254 | a | a | a | a | a | a | g | a | a | g | a |  |  |  |
|  | -143 | t | t | t | t | t | t | t | t | t | a | t |  |  |  |
|  | -104 | a | g | a | g | g | g | a | a | g | a | g |  |  |  |
|  | -61 | a | a | a | a | a | a | a | g | a | a | a |  |  |  |
| ***MKT1*** | 89 | **A** | **G** | **G** | **G** | **G** | **G** | **G** | **G** | **G** | **G** | **G** | 30 | D | G |
| ***MKT1*** | 213 | **G** | **G** | **G** | **G** | **G** | **G** | **A** | **G** | **G** | **G** | **G** | 71 | K | K |
| ***MKT1*** | 316 | **T** | **T** | **T** | **T** | **T** | **T** | **A** | **T** | **T** | **T** | **T** | 106 | S | T |
| ***MKT1*** | 345 | **C** | **C** | **C** | **C** | **C** | **T** | **C** | **C** | **C** | **C** | **C** | 115 | T | T |
| ***MKT1*** | 384 | **G** | **G** | **G** | **G** | **G** | **G** | **A** | **A** | **G** | **A** | **G** | 128 | L | L |
| ***MKT1*** | 462 | **A** | **A** | **A** | **A** | **A** | **A** | **A** | **A** | **A** | **G** | **A** | 154 | Q | Q |
| ***MKT1*** | 543 | **T** | **T** | **T** | **T** | **T** | **T** | **C** | **C** | **T** | **T** | **T** | 181 | H | H |
| ***MKT1*** | 978 | **A** | **A** | **A** | **A** | **A** | **A** | **G** | **G** | **A** | **G** | **A** | 326 | P | P |
| ***MKT1*** | 1010 | **T** | **T** | **T** | **T** | **T** | **T** | **T** | **T** | **T** | **C** | **T** | 337 | F | S |
| ***MKT1*** | 1050 | **G** | **G** | **G** | **G** | **G** | **G** | **G** | **G** | **G** | **A** | **G** | 350 | K | K |
| ***MKT1*** | 1295 | **C** | **C** | **C** | **G** | **G** | **G** | **C** | **C** | **C** | **C** | **C** | 432 | A | G |
| ***MKT1*** | 1308 | **A** | **A** | **A** | **A** | **A** | **A** | **G** | **G** | **A** | **G** | **A** | 436 | G | G |
| ***MKT1*** | 1358 | **A** | **G** | **G** | **G** | **G** | **G** | **G** | **G** | **G** | **G** | **G** | 453 | K | R |
| ***MKT1*** | 1572 | **A** | **A** | **A** | **A** | **A** | **A** | **G** | **G** | **A** | **G** | **A** | 524 | E | E |
| ***MKT1*** | 1590 | **C** | **C** | **C** | **C** | **C** | **C** | **T** | **T** | **C** | **T** | **C** | 530 | F | F |
| ***MKT1*** | 1710 | **C** | **C** | **C** | **C** | **C** | **C** | **T** | **T** | **C** | **T** | **C** | 570 | T | T |
| ***MKT1*** | 1731 | **A** | **A** | **A** | **A** | **A** | **A** | **G** | **G** | **A** | **G** | **A** | 577 | V | V |
| ***MKT1*** | 1782 | **G** | **G** | **G** | **G** | **G** | **G** | **A** | **A** | **G** | **A** | **G** | 594 | T | T |
| ***MKT1*** | 1800 | **C** | **C** | **C** | **C** | **C** | **C** | **A** | **A** | **C** | **A** | **C** | 600 | F | L |
| ***MKT1*** | 1828 | **A** | **A** | **A** | **A** | **A** | **A** | **C** | **C** | **A** | **C** | **A** | 610 | S | R |
| ***MKT1*** | 1830 | **T** | **T** | **T** | **T** | **T** | **T** | **T** | **G** | **T** | **T** | **T** | 610 | S | R |
| ***MKT1*** | 1969 | **G** | **G** | **G** | **G** | **G** | **G** | **G** | **A** | **G** | **G** | **G** | 657 | V | I |
| ***MKT1*** | 1972 | **C** | **C** | **C** | **C** | **C** | **C** | **C** | **C** | **C** | **T** | **C** | 658 | L | L |
| ***MKT1*** | 2001 | **T** | **T** | **T** | **T** | **T** | **T** | **C** | **T** | **T** | **C** | **T** | 667 | F | F |
| ***MKT1*** | 2040 | **T** | **T** | **T** | **T** | **T** | **T** | **C** | **T** | **T** | **C** | **T** | 680 | I | I |
| ***MKT1*** | 2094 | **A** | **A** | **G** | **A** | **A** | **A** | **A** | **A** | **A** | **A** | **A** | 698 | L | L |
| ***MKT1*** | 2430 | **T** | **T** | **T** | **T** | **T** | **T** | **T** | **C** | **T** | **T** | **T** | 810 | D | D |
|  | +49 | m | m | m | m | m | m | m | m | m | ∆ | m |  |  |  |
|  | +50 | c | c | c | c | c | c | c | t | c | c | c |  |  |  |
|  | +54 | a | t | a | t | t | t | a | a | t | a | t |  |  |  |
|  | +286 | a | a | a | a | a | a | a | g | a | g | a |  |  |  |
|  | -221 | a | ∆ | a | ∆ | ∆ | ∆ | a | a | a | a | a |  |  |  |
|  | -219 | t | t | T | t | t | t | c | t | t | t | t |  |  |  |
| ***END3*** | 802 | **C** | **T** | **T** | **T** | **T** | **T** | **T** | **T** | **T** | **T** | **T** | 268 | D | N |
| ***END3*** | 773 | **C** | **T** | **C** | **T** | **T** | **T** | **C** | **C** | **T** | **C** | **T** | 258 | S | N |
| ***END3*** | 735 | **G** | **G** | **G** | **G** | **G** | **G** | **G** | **T** | **G** | **G** | **G** | 245 | D | E |
| ***END3*** | 690 | **G** | **G** | **G** | **G** | **G** | **G** | **G** | **A** | **G** | **A** | **G** | 230 | S | S |
| ***END3*** | 663 | **T** | **C** | **T** | **C** | **C** | **C** | **T** | **T** | **C** | **T** | **C** | 221 | K | K |
| ***END3*** | 516 | **C** | **C** | **T** | **C** | **C** | **C** | **T** | **T** | **C** | **T** | **C** | 172 | K | K |
| ***END3*** | 302 | **T** | **T** | **T** | **T** | **T** | **T** | **T** | **T** | **T** | **T** | **T** | 101 | K | R |
| ***END3*** | 259 | **A** | **G** | **A** | **G** | **G** | **G** | **A** | **A** | **G** | **A** | **G** | 87 | L | L |
| ***END3*** | 21 | **A** | **A** | **A** | **A** | **A** | **A** | **A** | **A** | **A** | **G** | **A** | 7 | F | F |
|  | -48 | c | g | c | c | c | c | c | c | c | c | c |  |  |  |
|  | -114 | t | t | t | t | t | t | t | t | t | a | t |  |  |  |

**REFERENCES:**

1. Steinmetz LM, Sinha H, Richards DR, Spiegelman JI, Oefner PJ, et al. (2002) Dissecting the architecture of a quantitative trait locus in yeast. Nature 416: 326-330.
