# Supplementary material for: Complex Genetic Interactions in a Quantitative Trait Locus
Source: PLoS Genet. 2006 Feb 3;2(2):e13. doi: 10.1371/journal.pgen.0020013 (PMC1359075; doi:10.1371/journal.pgen.0020013)
Supplement: Supplementary file 4 [file pgen.0020013.st004.doc]

**Table S4:** List of strains used in this study.

| **Strain name** | Derivative of | Genotype | **Reference** |
| --- | --- | --- | --- |
| S1 | S288c | *MATα gal2* | This study |
| S1001 | S1 | *ho/ho* | [1] |
| S1002 | S25 | *lys2/lys2* | [1] |
| S1003 | S96 | *lys5/lys5* | [1] |
| S1019 | S1003 | *END3-288/end3∆::Nat* | [1] |
| S1022 | S1002 | END3-288/end3∆::Hyg | [1] |
| S1023 | S1002 | *MKT1-288/mkt1∆::Hyg* | [1] |
| S1027 | S1003 | *MKT1-288/mkt1∆::Nat* | [1] |
| S1029 | S1001 | *ho/ho∆::Nat* | [1] |
| S103 | S94 | *MATa ho∆::KanMX3* | This study |
| S1036 | S103 | *MATa ho∆::Hyg* | This study |
| S1038 | S1002 | *ARP2-288/arp2∆::Hyg* | This study |
| S1046 | S1003 | *RHO2-288/rho2∆::Nat* | [1] |
| S1054 | S1002 | *PAN1-288/pan1∆::Hyg* | This study |
| S1056 | S1002 | *RHO2-288/rho2∆::Hyg* | [1] |
| S1149 | S1003 | *END3-288/end3∆::Nat* | This study |
| S1162 | S1159 | *MATα end3(773-802)∆::loxP-Kan-loxP* | This study |
| S1288 | S288c | *MATα lys5::loxP* | [2] |
| S1534 | S94 | *MATa mkt1(1339-1379)∆::Hyg/T-urf13MX4* | This study |
| S1537 | S1534 | *MATa MKT1-288(453R)* | This study |
| S1538 | S90 | *MATα rho2∆::Nat* | This study |
| S1544 | S90 | *MATα rho2∆::Hyg* | This study |
| S1548 | S94 | MATa rho2∆::Hyg | This study |
| S1549 | S94 | MATa rho2∆::Hyg | This study |
| S1564 | S90 | *MAT**α mkt1∆::Nat* | This study |
| S1570 | S90 | *MATα end3∆::Hyg* | This study |
| S1572 | S94 | *MATa end3∆::Hyg* | This study |
| S1573 | S94 | *MATa end3∆::Hyg* | This study |
| S1576 | S94 | *MATa end3∆::Nat* | This study |
| S1577 | S94 | *MATa end3∆::Nat* | This study |
| S1578 | S94 | MATa mkt1∆::Hyg | This study |
| S1578 | S94 | *MATa mkt1∆::Hyg* | This study |
| S1579 | S94 | *MATa mkt1∆::Hyg* | This study |
| S1580 | YBN501 | MATα RHO2-288(91F)* | This study |
| S1583 | YBN502 | *MATa RHO2-145(91C)** | This study |
| S1585 | S1548×S1580 | RHO2-288/rho2∆::Hyg | This study |
| S1593 | S1288 | *MATα mkt1∆(69-1379)::LYS5*MX4 | This study |
| S1602 | S90 | *MATα end3∆::Kan* | This study |
| S1612 | S94 | *MATa mkt1∆::Kan* | This study |
| S1613 | S94 | *MATa mkt1∆::Kan* | This study |
| S1614 | S90 | *MATα rho2∆::Kan* | This study |
| S1615 | S94 | MATa rho2∆::Kan | This study |
| S1617 | S1583×S1614 | *RHO2-145/rho2∆::Kan* | This study |
| S1622 | S1162 | *MATa END3-288(258S 268D)** | This study |
| S1623 | S1162 | *MATa END3-288(258S 268D)** | This study |
| S1624 | S1162 | *MATa END3-145(258N 268N)** | This study |
| S1625 | S1162 | *MATa END3-145(258N 268N)** | This study |
| S1630 | S1570×S1622 | *END3-288/end3∆::Hyg* | This study |
| S1631 | S1570×S1623 | *END3-288/end3∆::Hyg* | This study |
| S1632 | S1602×S1624 | *END3-145/end3∆::Kan* | This study |
| S1633 | S1602×S1625 | *END3-145/end3∆::Kan* | This study |
| S1642 | YBN501 | MATα RHO2-288(91F)* | This study |
| S1644 | YBN501 | MATα RHO2-145(91C)* | This study |
| S1645 | YBN502 | *MATa RHO2-145(91C)** | This study |
| S1648 | S1127 | *end3∆::loxP/end3∆::loxP lys2* | This study |
| S1649 | S1127 | *end3∆::loxP/end3∆::loxP lys2* | This study |
| S1650 | S1593 | MATα MKT1-288(30D 453K)* | This study |
| S1651 | S1593 | *MATα MKT1-288(30D 453K)** | This study |
| S1652 | S1593 | *MATα MKT1-145(30G 453R)** | This study |
| S1653 | S1593 | *MATα MKT1-145(30G 453R)** | This study |
| S1654 | S1650×S1578 | MKT1-288/mkt1∆::Hyg | This study |
| S1655 | S1651×S1579 | *MKT1-288/mkt1∆::Hyg* | This study |
| S1656 | S1652×S1612 | *MKT1-145/mkt1∆::Kan* | This study |
| S1657 | S1653×S1613 | *MKT1-145/mkt1∆::Kan* | This study |
| S1658 | S1646 | *RHO2-288/rho2∆::Kan mkt1∆::loxP/mkt1∆::loxP lys2* | This study |
| S1659 | S1647 | *RHO2-288/rho2∆::Kan mkt1∆::loxP/mkt1∆::loxP lys2* | This study |
| S1660 | S1642×S1549 | RHO2-288/rho2∆::Hyg | This study |
| S1661 | S1644×S1615 | *RHO2-145/rho2∆::Kan* | This study |
| S1662 | S94 | *MATa ho/ho∆::Hyg* | This study |
| S1663 | S94 | *MATa ho/ho∆::Hyg* | This study |
| S1671 | S1002 | *APM3-288/apm3∆::Hyg* | This study |
| S1673 | S1003 | *APM3-288/apm3∆::Nat* | This study |
| S1675 | S1002 | *RVS161-288/rvs161∆::Hyg* | This study |
| S1676 | S1002 | *RVS167-288/rvs167∆::Hyg* | This study |
| S1678 | S1003 | *RVS167-288/rvs167∆::Nat* | This study |
| S1682 | S1003 | *RVS161-288/rvs161∆::Nat* | This study |
| S1696 | S1564 | *MATα mkt1∆::Nat rho2∆::loxP-Kan-loxP* | This study |
| S1697 | S1578 | *MATa mkt1∆::Hyg rho2∆::loxP-Kan-loxP* | This study |
| S19 | S1 | *MATα ura3* | This study |
| S25 | S1 | *MATα lys2-1* | This study |
| S3207 | S795 | *MATα fcy1∆ lys2-1 RHO2-288*-3’utr*∆::FCY1/Nat* | This study |
| S3209 | S3207 | *MATα fcy1∆ lys2-1 RHO2-288*-3’UTR-145* | This study |
| S3210 | S3207 | *MATα fcy1∆ lys2-1 RHO2-288*-3’UTR-145* | This study |
| S529 | S90 | *MATα ura3∆::Kan* | This study |
| S775 | S96 | *MATa lys5 fcy1∆* | This study |
| S795 | S775×S25 | *MATα fcy1∆ lys2-1* | This study |
| S822 | S91 | *MAT**α rho2(216-324bp)∆::Hyg/T-urf13MX4* | This study |
| S823 | S90 | *MAT**α rho2(216-324bp)∆::Hyg/T-urf13MX4* | This study |
| S824 | S94 | *MATa rho2(216-324bp)∆::Hyg/T-urf13MX4* | This study |
| S90 | S1 | *MATα GAL2* | This study |
| S91 | S1 | *MATα GAL2* | This study |
| S94 | S288c | *MATa* | This study |
| S96 | S288c | *MATa lys5* | This study |
| XHS104 | YJM155×S1027 | *MKT1-145/mkt1∆::Nat* | [1] |
| XHS107 | YJM385×S1022 | END3-145/end3∆::Hyg | [1] |
| XHS108 | YJM385×S1023 | *MKT1-145/mkt1∆::Hyg* | [1] |
| XHS111 | YHS131×S1002 | *end3∆::Nat*/*END3-288* | [1] |
| XHS114 | YHS167×S1002 | *mkt1∆::Nat*/*MKT1-288* | [1] |
| XHS118 | YHS172×S1003 | *end3∆::Hyg*/*END3-288* | [1] |
| XHS119 | YHS166×S1003 | mkt1∆::Hyg/MKT-288 | [1] |
| XHS122 | YJM155×S1019 | *END3-145/end3∆::Nat* | [1] |
| XHS123 | YJM155×S529 | *MKT1-145*/*MKT1-288 END3-145*/*END3-288*  *RHO2-145*/*RHO2-288 HO/ho∆::Kan* | [1] |
| XHS128 | YJM385×S1038 | *ARP2-145/arp2∆::Hyg* | This study |
| XHS130 | YJM385×S1054 | *PAN1-145/pan1∆::Hyg* | This study |
| XHS131 | YJM385×S1056 | *RHO2-145/rho2∆::Hyg* | [1] |
| XHS132 | YJM155×S1046 | *RHO2-145/rho2∆::Nat* | [1] |
| XHS138 | YHS229×S1003 | *rho2∆::Hyg*/*RHO2-288* | [1] |
| XHS140 | YHS251×S1002 | *rho2∆::Nat*/*RHO2-288* | [1] |
| XHS142 | YHS259×S1002 | *arp2∆::Nat*/*ARP2-288* | This study |
| XHS148 | YHS261×S1002 | *pan1∆::Nat*/*PAN1-288* | This study |
| XHS211 | YJM270×S288c | *mkt1∆::Nat/MKT1-288* | This study |
| XHS215 | YJM421×S288c | *END3-421/end3∆::Hyg* | This study |
| XHS217 | YJM421×S288c | *RHO2-421/rho2∆::Hyg* | This study |
| XHS229 | YJM339×S288c | *END3-339/end3∆::Nat* | This study |
| XHS232 | YJM339×S288c | *RHO2-339/rho2∆::Hyg* | This study |
| XHS236 | YJM270×S288c | *END3-270/end3∆::Nat* | This study |
| XHS237 | YJM280×S288c | *MKT1-280/mkt1∆::Nat* | This study |
| XHS239 | YJM326×S288c | *MKT1-326/mkt1∆::Nat* | This study |
| XHS240 | YJM326×S288c | *RHO2-326/rho2∆::Nat* | This study |
| XHS241 | YJM326×S288c | *END3-326/end3∆::Nat* | This study |
| XHS242 | YJM320×S288c | *MKT1-320/mkt1∆::Nat* | This study |
| XHS244 | YJM320×S288c | *END3-320/end3∆::Nat* | This study |
| XHS245 | YJM280×S288c | *END3-280/end3∆::Nat* | This study |
| XHS246 | YJM421×S288c | *MKT1-421/mkt1∆::Nat* | This study |
| XHS247 | YJM421×S288c | *RHO2-421/rho2∆::Nat* | This study |
| XHS248 | YJM421×S288c | *END3-421/end3∆::Hyg* | This study |
| XHS249 | YJM627×S288c | *MKT1-627/mkt1∆::Nat* | This study |
| XHS253 | YJM270×S288c | *MKT1-270/mkt1∆::Nat* | This study |
| XHS254 | YJM270×S288c | *END3-270/end3∆::Hyg* | This study |
| XHS255 | YJM270×S288c | *MKT1-270/mkt1∆::Hyg* | This study |
| XHS256 | YJM270×S288c | *RHO2-270/rho2∆::Hyg* | This study |
| XHS260 | YJM339×S288c | *END3-339/end3∆::Hyg* | This study |
| XHS264 | YJM326×S288c | *mkt1∆::Hyg/MKT1-288* | This study |
| XHS265 | YJM320×S288c | *end3∆::Hyg/END3-288* | This study |
| XHS266 | YJM326×S288c | *end3∆::Hyg/END3-288* | This study |
| XHS268 | YJM326×S288c | *rho2∆::Hyg/RHO2-288* | This study |
| XHS271 | YJM1129×S288c | *rho2∆::Hyg/RHO2-288* | This study |
| XHS272 | YJM1129×S288c | *mkt1∆::Hyg/MKT1-288* | This study |
| XHS273 | YJM320×S288c | *end3∆::Nat/END3-288* | This study |
| XHS275 | YJM326×S288c | *end3∆::Nat/END3-288* | This study |
| XHS278 | YJM326×S288c | *rho2∆::Nat/RHO2-288* | This study |
| XHS282 | YJM270×S288c | *RHO2-270/rho2∆::Nat* | This study |
| XHS284 | YJM339×S288c | *RHO2-339/rho2∆::Nat* | This study |
| XHS285 | YJM1129×S288c | *RHO2-1129/rho2∆::Nat* | This study |
| XHS286 | YJM1129×S288c | *END3-1129/end3∆::Nat* | This study |
| XHS287 | YJM1129×S288c | *MKT1-1129/mkt1∆::Nat* | This study |
| XHS288 | YJM1129×S288c | *rho2∆::Nat/RHO2-288* | This study |
| XHS289 | YJM1129×S288c | *mkt1∆::Nat/MKT1-288* | This study |
| XHS290 | YJM1129×S288c | *end3∆::Nat/END3-288* | This study |
| XHS293 | YJM280×S288c | *mkt1∆::Nat/MKT1-288* | This study |
| XHS296 | YJM421×S288c | *mkt1∆::Nat/MKT1-288* | This study |
| XHS297 | YJM421×S288c | *rho2∆::Nat/RHO2-288* | This study |
| XHS298 | YJM421×S288c | *mkt1∆::Hyg/MKT1-288* | This study |
| XHS299 | YJM421×S288c | *end3∆::Hyg/END3-288* | This study |
| XHS300 | YJM421×S288c | *rho2∆::Hyg/RHO2-288* | This study |
| XHS301 | YJM421×S288c | *end3∆::Nat/END3-288* | This study |
| XHS315 | YJM326×S288c | *mkt1∆::Nat/MKT1-288* | This study |
| XHS317 | YJM1129×S288c | *end3∆::Hyg/END3-288* | This study |
| XHS323 | YJM326×S288c | *MKT1-326/mkt1∆::Hyg* | This study |
| XHS324 | YJM280×S288c | *END3-280/end3∆::Hyg* | This study |
| XHS325 | YJM280×S288c | *MKT1-280/mkt1∆::Hyg* | This study |
| XHS327 | YJM320×S288c | *MKT1-320/mkt1∆::Hyg* | This study |
| XHS328 | YJM326×S288c | *END3-326/end3∆::Hyg* | This study |
| XHS329 | YJM1129×S288c | *END3-1129/mkt1∆::Hyg* | This study |
| XHS330 | YJM1129×S288c | *MKT1-1129/mkt1∆::Hyg* | This study |
| XHS331 | YJM1076×S288c | *END3-1076/end3∆::Hyg* | This study |
| XHS332 | YJM1076×S288c | *MKT1-1076/mkt1∆::Hyg* | This study |
| XHS334 | YJM320×S288c | *END3-320/end3∆::Hyg* | This study |
| XHS335 | YJM270×S288c | *end3∆::Hyg/END3-288* | This study |
| XHS336 | YJM339×S288c | *end3∆::Hyg/END3-288* | This study |
| XHS339 | YJM1076×S288c | *end3∆::Hyg/END3-288* | This study |
| XHS340 | YJM280×S288c | *end3∆::Hyg/END3-288* | This study |
| XHS343 | YJM627×S288c | *end3∆::Hyg/END3-288* | This study |
| XHS344 | YJM627×S288c | *END3-627/end3∆::Hyg* | This study |
| XHS345 | YJM627×S288c | *MKT1-627/mkt1∆::Hyg* | This study |
| XHS346 | YJM627×S288c | *RHO2-627/rho2∆::Hyg* | This study |
| XHS356 | YJM326×S288c | *RHO2-326/rho2∆::Hyg* | This study |
| XHS358 | YJM1076×S288c | *RHO2-1076/rho2∆::Hyg* | This study |
| XHS359 | YJM1129×S288c | *RHO2-1129/rho2∆::Hyg* | This study |
| XHS363 | YJM627×S288c | *MKT1-627/mkt1∆::Nat* | This study |
| XHS364 | YJM627×S288c | *RHO2-627/rho2∆::Nat* | This study |
| XHS365 | YJM627×S288c | *END3-627/end3∆::Nat* | This study |
| XHS370 | YJM270×S288c | *mkt1∆::Hyg/MKT1-288* | This study |
| XHS376 | YJM1076×S288c | *rho2∆::Hyg/RHO2-288* | This study |
| XHS377 | YJM270×S288c | *end3∆::Nat/END3-288* | This study |
| XHS386 | YJM627×S288c | *rho2∆::Nat/RHO2-288* | This study |
| XHS387 | YJM1076×S288c | *mkt1∆::Nat/MKT1-288* | This study |
| XHS389 | YJM1076×S288c | *rho2∆::Nat/RHO2-288* | This study |
| XHS390 | YJM627×S288c | *end3∆::Nat/END3-288* | This study |
| XHS392 | YJM339×S288c | *end3∆::Nat/END3-288* | This study |
| XHS393 | YJM339×S288c | *HO/ho∆::Kan* | This study |
| XHS395 | YJM280×S288c | *HO/ho∆::Kan* | This study |
| XHS396 | YJM326×S288c | *HO/ho∆::Kan* | This study |
| XHS397 | YJM627×S288c | *HO/ho∆::Kan* | This study |
| XHS398 | YJM1129×S288c | *HO/ho∆::Kan* | This study |
| XHS399 | YJM1076×S288c | *HO/ho∆::Kan* | This study |
| XHS400 | YJM270×S288c | *HO/ho∆::Kan* | This study |
| XHS401 | YJM320×S288c | *HO/ho∆::Kan* | This study |
| XHS402 | YJM280×S288c | *rho2∆::Nat/RHO2-288* | This study |
| XHS403 | YJM270×S288c | *rho2∆::Nat/RHO2-288* | This study |
| XHS404 | YJM320×S288c | *mkt1∆::Nat/MKT1-288* | This study |
| XHS405 | YJM339×S288c | *rho2∆::Nat/RHO2-288* | This study |
| XHS406 | YJM320×S288c | *rho2∆::Nat/RHO2-288* | This study |
| XHS407 | YJM627×S288c | *mkt1∆::Nat/MKT1-288* | This study |
| XHS408 | YJM280×S288c | *end3∆::Nat/END3-288* | This study |
| XHS409 | YJM320×S288c | *rho2∆::Hyg/RHO2-288* | This study |
| XHS410 | YJM627×S288c | *mkt1∆::Hyg/MKT1-288* | This study |
| XHS411 | YJM339×S288c | *rho2∆::Hyg/RHO2-288* | This study |
| XHS412 | YJM339×S288c | *mkt1∆::Hyg/MKT1-288* | This study |
| XHS413 | YJM320×S288c | *mkt1∆::Hyg/MKT1-288* | This study |
| XHS414 | YJM270×S288c | *rho2∆::Hyg/RHO2-288* | This study |
| XHS415 | YJM280×S288c | *end3∆::Hyg/END3-288* | This study |
| XHS417 | YJM627×S288c | *rho2∆::Hyg/RHO2-288* | This study |
| XHS418 | YJM280×S288c | *rho2∆::Hyg/RHO2-288* | This study |
| XHS420 | YJM1076×S288c | *MKT1-1076/mkt1∆::Nat* | This study |
| XHS421 | YJM1076×S288c | *END3-1076/end3∆::Nat* | This study |
| XHS422 | YJM1076×S288c | *RHO2-1076/rho2∆::Nat* | This study |
| XHS425 | YJM1076×S288c | *end3∆::Hyg/END3-288* | This study |
| XHS526 | YHS750×S1622 | *end3∆::Kan*/*END3-288* | This study |
| XHS527 | YHS751×S1623 | *end3∆::Kan*/*END3-288* | This study |
| XHS532 | YHS806×S1036 | *MKT1-288/MKT1-288 ho∆::Hyg* | This study |
| XHS533 | YHS807×S1036 | *MKT1-288/MKT1-288 ho∆::Hyg* | This study |
| XHS534 | YHS485×S1624 | *END3-145/END3-145 ho∆::Kan* | This study |
| XHS535 | YHS491×S1625 | *END3-145/END3-145 ho∆::Kan* | This study |
| XHS536 | YHS778×S1036 | *END3-288/END3-288 ho∆::Hyg* | This study |
| XHS537 | YHS779×S1036 | *END3-288/END3-288 ho∆::Hyg* | This study |
| XHS538 | YHS722×S1036 | *RHO2-288/RHO2-288 ho∆::Hyg* | This study |
| XHS541 | YHS485×S1583 | *RHO2-145/RHO2-145 ho∆::Kan* | This study |
| XHS542 | YHS491×S1645 | *RHO2-145/RHO2-145 ho∆::Kan* | This study |
| XHS543 | YHS490×S1652 | *MKT1-145/MKT1-145 ho∆::Kan* | This study |
| XHS544 | YHS490×S1653 | *MKT1-145/MKT1-145 ho∆::Kan* | This study |
| XHS545 | YHS816×S1658 | *MKT1-145/mkt1∆::loxP  end3∆::loxP/END3-288  RHO2-145/rho2∆::Kan* | This study |
| XHS546 | YHS817×S1659 | *MKT1-145/mkt1∆::loxP  end3∆::loxP/END3-288  RHO2-145/rho2∆::Kan* | This study |
| XHS547 | YHS661×S1648 | *mkt1∆::loxP/MKT1-288  END3-145/end3∆::loxP  rho2∆::loxP/RHO2-288* | This study |
| XHS548 | YHS662×S1649 | *mkt1∆::loxP/MKT1-288  END3-145/end3∆::loxP  rho2∆::loxP/RHO2-288* | This study |
| XHS551 | YHS823×S1652 | *MKT1-145/MKT1-145 ho∆::Kan* | This study |
| XHS552 | YHS824×S1653 | *MKT1-145/MKT1-145 ho∆::Kan* | This study |
| XHS553 | YHS825×S1624 | *END3-145/END3-145 ho∆::Kan* | This study |
| XHS554 | YHS826×S1625 | *END3-145/END3-145 ho∆::Kan* | This study |
| XHS555 | YHS778×S1662 | *END3-288/END3-288 ho∆::Hyg* | This study |
| XHS556 | YHS779×S1663 | *END3-288/END3-288 ho∆::Hyg* | This study |
| XHS557 | YHS806×S1662 | *MKT1-288/MKT1-288 ho∆::Hyg* | This study |
| XHS558 | YHS807×S1663 | *MKT1-288/MKT1-288 ho∆::Hyg* | This study |
| XHS559 | YHS719×S1564 | *mkt1∆::Nat*/*mkt1∆::Nat* | This study |
| XHS560 | YHS720×S1658 | *mkt1∆::Hyg*/*mkt1∆::Hyg* | This study |
| XHS561 | YHS736×S1544 | *rho2∆::Hyg*/*rho2∆::Hyg* | This study |
| XHS562 | YHS718×S1538 | *rho2∆::Nat*/*rho2∆::Nat* | This study |
| XHS566 | YHS189×S1149 | *mkt1∆::Hyg*/*MKT1-288 END3-145*/*end3∆::Nat* | This study |
| XHS567 | YHS167×S1022 | *mkt1∆::Nat*/*MKT1-288 END3-145*/*end3∆::Hyg* | This study |
| XHS568 | YHS131×S1023 | *MKT1-145*/*mkt1∆::Hyg end3∆::Nat*/*END3-288* | This study |
| XHS569 | YHS172×S1027 | *MKT1-145*/*mkt1∆::Nat end3∆::Hyg*/*END3-288* | This study |
| XHS570 | YHS229×S1149 | *rho2∆::Hyg*/*RHO2-288 END3-145*/*end3∆::Nat* | This study |
| XHS571 | YHS251×S1022 | *rho2∆::Nat*/*RHO2-288 END3-145*/*end3∆::Hyg* | This study |
| XHS572 | YHS131×S1056 | *RHO2-145*/*rho2∆::Hyg end3∆::Nat*/*END3-288* | This study |
| XHS573 | YHS172×S1046 | *RHO2-145*/*rho2∆::Nat end3∆::Hyg*/*END3-288* | This study |
| XHS578 | YHS838×S1003 | *apm3∆::Hyg*/*APM3-288* | This study |
| XHS580 | YHS840×S1002 | *apm3∆::Nat*/*APM3-288* | This study |
| XHS582 | YJM385×S1671 | *APM3-145/apm3∆::Hyg* | This study |
| XHS584 | YJM155×S1673 | *APM3-145/apm3∆::Nat* | This study |
| XHS586 | YHS842×S1003 | *rvs161∆::Hyg*/*RVS161-288* | This study |
| XHS587 | YHS844×S1002 | *rvs161∆::Nat*/*RVS161-288* | This study |
| XHS588 | YHS846×S1003 | *rvs167∆::Hyg*/*RVS167-288* | This study |
| XHS589 | YHS848×S1002 | *rvs167∆::Nat*/*RVS167-288* | This study |
| XHS590 | YJM155×S1678 | *RVS167-145/rvs167∆::Nat* | This study |
| XHS591 | YJM385×S1675 | *RVS161-145/rvs161∆::Hyg* | This study |
| XHS592 | YJM385×S1676 | *RVS167-145/rvs167∆::Hyg* | This study |
| XHS593 | YJM155×S1682 | *RVS161-145/rvs161∆::Nat* | This study |
| XHS647 | YHS894×S90 | *∆::Nat/MKT1-288 ∆::Hyg/RHO2-288* | This study |
| XHS648 | YHS895×S90 | *∆::Hyg/MKT1-288 ∆::Nat/RHO2-288* | This study |
| XHS649 | YHS696×S1696 | *MKT1-145/∆::Nat  RHO2-145/∆::loxP-Kan-loxP* | This study |
| XHS650 | YHS605×S1697 | *MKT1-145/∆::Hyg  RHO2-145/∆::loxP-Kan-loxP* | This study |
| XHS656 | YHS605×S747 | *HO/ho∆::Hyg* | This study |
| XHS657 | YHS605×S748 | *HO/ho∆::Nat* | This study |
| XHS658 | YHS718×S3209 | *rho2∆::Nat/RHO2-288*-3’UTR-145* | This study |
| XHS659 | YHS737×S3210 | *rho2∆::Hyg/RHO2-288*-3’UTR-145* | This study |
| XHS660 | YHS689×S1566 | *MKT1-145(30D)/mkt1∆::Nat* | This study |
| XHS661 | YHS690×S1578 | *MKT1-145(30G)/mkt1∆::Hyg* | This study |
| XHS662 | YHS691×S1537 | *MKT1-288(453R)/mkt1∆::Hyg* | This study |
| XHS663 | YHS719×S90 | *MKT1-288(453K)/mkt1∆::Nat* | This study |
| XHS664 | YHS697×S1572 | *END3-145(258S)/end3∆::Hyg* | This study |
| XHS665 | YHS700×S1576 | *END3-145(258N)/end3∆::Nat* | This study |
| XHS666 | YHS698×S1572 | *END3-145(268D)/end3∆::Hyg* | This study |
| XHS667 | YHS701×S1576 | *END3-145(268N)/end3∆::Nat* | This study |
| XHS668 | YHS699×S1573 | *END3-145(268D)/end3∆::Hyg* | This study |
| XHS669 | YHS702×S1577 | *END3-145(268N)/end3∆::Nat* | This study |
| XRP055 | YJM421×S288c | *HO/ho∆::Hyg* | This study |
| YAG040 | YJM145 | *HO/ho∆::Hyg* | [3] |
| YBN504 | YHS605 | *MATα rho2(216-324bp)∆::Hyg/T-urf13MX4* | This study |
| YBN505 | YHS607 | *MATα rho2(216-324bp)∆::Hyg/T-urf13MX4* | This study |
| YHS131 | YJM385 | *END3-145/end3∆::Nat* | [1] |
| YHS166 | YJM155 | MKT1-145/mkt1∆::Hyg | [1] |
| YHS167 | YJM385 | *MKT1-145/mkt1∆::Nat* | [1] |
| YHS172 | YJM155 | *END3-145/end3∆::Hyg* | [1] |
| YHS189 | YJM145 | *MKT1-145/mkt1∆::Hyg* | This study |
| YHS200 | YJM145 | *mkt1∆::Kan/mkt1∆::Kan* | This study |
| YHS208 | YJM145 | *HO/ho∆::Kan* | This study |
| YHS222 | YJM145 | END3-145/end3∆::Nat | This study |
| YHS229 | YJM155 | *RHO2-145/rho2∆::Hyg* | [1] |
| YHS251 | YJM385 | *RHO2-145/rho2∆::Nat* | [1] |
| YHS259 | YJM385 | *ARP2-145/arp2∆::Nat* | This study |
| YHS261 | YJM385 | *PAN1-145/pan1∆::Nat* | This study |
| YHS346 | YJM145 | *end3∆::Hyg/end3∆::Hyg* | This study |
| YHS349 | YJM145 | *end3∆::Nat/end3∆::Nat* | This study |
| YHS356 | YJM145 | *rho2∆::Nat/rho2∆::Nat* | This study |
| YHS437 | YJM145 | *FLO8/flo8∆::loxP HO/ho∆::loxP-Kan-loxP* | This study |
| YHS485 | YHS437 | *MATα flo8∆::loxP ho∆::loxP-Kan-loxP* | This study |
| YHS490 | YHS437 | *MATα flo8∆::loxP ho∆::loxP-Kan-loxP* | This study |
| YHS491 | YHS437 | *MATα flo8∆::loxP ho∆::loxP-Kan-loxP* | This study |
| YHS605 | YHS437 | *MATα ho∆::loxP flo8∆::loxP* | This study |
| YHS607 | YJM145 | *MATα ho∆::loxP flo8∆::loxP* | This study |
| YHS616 | YJM280 | *HO/ho∆::Hyg* | This study |
| YHS629 | YJM339 | *HO/ho∆::Hyg* | This study |
| YHS631 | YJM627 | *HO/ho∆::Hyg* | This study |
| YHS633 | YHS605 | *MATα* *ho∆::loxP flo8∆::loxP lys5∆::loxP* | This study |
| YHS661 | YJM385 | *mkt1∆::loxP/mkt1∆::loxP rho2∆::Hyg/rho2∆::Hyg* | This study |
| YHS662 | YJM385 | *mkt1∆::loxP/mkt1∆::loxP rho2∆::Hyg/rho2∆::Hyg* | This study |
| YHS667 | YJM320 | *HO/ho∆::Hyg* | This study |
| YHS669 | YJM326 | *HO/ho∆::Hyg* | This study |
| YHS671 | YJM1129 | *HO/ho∆::Hyg* | This study |
| YHS673 | YJM270 | *HO/ho∆::Hyg* | This study |
| YHS674 | YJM1076 | *HO/ho∆::Hyg* | This study |
| YHS676 | YHS605 | *MATα mkt1(69-110)∆::Hyg/T-urf13MX4* | This study |
| YHS687 | YHS607 | *MATα end3(773-809bp)∆::loxP-Kan-loxP* | This study |
| YHS688 | YHS607 | *MATα end3(773-809bp)∆::loxP-Kan-loxP* | This study |
| YHS689 | YHS676 | *MATα MKT1-145(30D)* | This study |
| YHS690 | YHS676 | *MATα MKT1-145(30G)* | This study |
| YHS691 | YHS605 | *MATα mkt1(69-1379)∆::Hyg* | This study |
| YHS695 | YHS437 | *MATa ho∆::loxP flo8∆::loxP* | This study |
| YHS696 | YHS437 | *MATa ho∆::loxP flo8∆::loxP* | This study |
| YHS697 | YHS688 | *MATα END3-145(258S)* | This study |
| YHS698 | YHS688 | *MATα END3-145(268D)* | This study |
| YHS699 | YHS688 | *MATα END3-145(268D)* | This study |
| YHS700 | YHS688 | *MATα END3-145(258N)* | This study |
| YHS701 | YHS688 | *MATα END3-145(258N 268N)* | This study |
| YHS702 | YHS688 | *MATα END3-145(258N 268N)* | This study |
| YHS718 | YHS695 | *MATa rho2∆::Nat* | This study |
| YHS719 | YHS695 | *MATa mkt1∆::Nat* | This study |
| YHS720 | YHS695 | *MATa mkt1∆::Hyg* | This study |
| YHS721 | YHS695 | MATa mkt1∆::Hyg | This study |
| YHS722 | YBN504 | MATα RHO2-288(91F)* | This study |
| YHS723 | YBN504 | MATα RHO2-145(91C)* | This study |
| YHS731 | YHS633 | MATα mkt1(69-1379 bp)∆::LYS5MX4 | This study |
| YHS733 | YHS696 | *MATa end3∆::Hyg* | This study |
| YHS736 | YHS696 | *MATa end3∆::Hyg* | This study |
| YHS737 | YHS696 | *MATa rho2∆::Hyg* | This study |
| YHS738 | YHS722×YHS736 | *rho2∆::Hyg*/*RHO2-288* | This study |
| YHS750 | YHS605 | MATα end3∆::Kan | This study |
| YHS751 | YHS605 | MATα end3∆::Kan | This study |
| YHS752 | YHS696 | *MATa end3∆::Kan* | This study |
| YHS768 | YHS696 | MATa rho2∆::Kan | This study |
| YHS769 | YHS723×YHS768 | *RHO2-145/rho2∆::Kan* | This study |
| YHS770 | YJM145 | *mkt1∆::Nat/mkt1∆::Nat* | This study |
| YHS778 | YHS688 | *MATα END3-288(258S 268D)** | This study |
| YHS779 | YHS688 | *MATα END3-288(258S 268D)** | This study |
| YHS782 | YHS701×YHS752 | *END3-145/end3∆::Kan* | This study |
| YHS783 | YHS702×YHS752 | *END3-145/end3∆::Kan* | This study |
| YHS785 | YJM145 | *rho2∆::Kan/rho2∆::Kan* | This study |
| YHS805 | YHS731 | *MATα MKT1-145(30D 453R)** | This study |
| YHS806 | YHS731 | *MATα MKT1-288(30D 453K)** | This study |
| YHS807 | YHS731 | *MATα MKT1-288(30D 453K)** | This study |
| YHS810 | YHS696 | *MATa mkt1∆Hyg* | This study |
| YHS814 | YHS805×YHS808 | *MKT1-145/mkt1∆::Kan* | This study |
| YHS815 | YHS805×YHS809 | *MKT1-145/mkt1∆::Kan* | This study |
| YHS816 | YHS433 | *end3∆::loxP/end3∆::loxP* | This study |
| YHS817 | YHS433 | *end3∆::loxP/end3∆::loxP* | This study |
| YHS818 | YHS720×YHS806 | *mkt1∆::Hyg*/*MKT1-288* | This study |
| YHS819 | YHS721×YHS807 | *mkt1∆::Hyg*/*MKT1-288* | This study |
| YHS820 | YHS733×YHS778 | *end3∆::Hyg*/*END3-288* | This study |
| YHS821 | YHS733×YHS779 | *end3∆::Hyg*/*END3-288* | This study |
| YHS822 | YHS722×YHS737 | *rho2∆::Hyg*/*RHO2-288* | This study |
| YHS823 | YHS437 | *MATa flo8∆::loxP ho∆::loxP-Kan-loxP* | This study |
| YHS824 | YHS437 | *MATa flo8∆::loxP ho∆::loxP-Kan-loxP* | This study |
| YHS825 | YHS437 | *MATα flo8∆::loxP ho∆::loxP-Kan-loxP* | This study |
| YHS826 | YHS437 | *MATα flo8∆::loxP ho∆::loxP-Kan-loxP* | This study |
| YHS827 | YAG129 | END3-145/end3∆::Kan | This study |
| YHS838 | YJM155 | *APM3-145/apm3∆::Hyg* | This study |
| YHS840 | YJM385 | *APM3-145/apm3∆::Nat* | This study |
| YHS842 | YJM155 | *RVS161-145/rvs161∆::Hyg* | This study |
| YHS844 | YJM385 | *RVS161-145/rvs161∆::Nat* | This study |
| YHS846 | YJM155 | *RVS167-145/rvs167∆::Hyg* | This study |
| YHS848 | YJM385 | *RVS167-145/rvs167∆::Nat* | This study |
| YHS894 | YHS719 | *MATa mkt1∆::Nat rho2∆::Hyg* | This study |
| YHS895 | YHS810 | *MATa mkt1∆::Hyg rho2∆::Nat* | This study |
| YJM1076 | ATCC 204722 | *HO/HO* | This study |
| YJM1084 | YJM1076 | *lys2/lys2* | This study |
| YJM1129 | Segregant of distillery strain NCMH125 | *HO/HO* | This study |
| YJM1183 | YJM1129 | *lys2/lys2* | This study |
| YJM145 | Segregant of a clinical isolate | *HO/HO* | [4] |
| YJM155 | YJM145 | *lys2/lys2* | [4] |
| YJM270 | Segregant of wine strain CBS2807 | *HO/HO* | This study |
| YJM280 | Segregant of a clinical isolate | *HO/HO* | [4] |
| YJM301 | YJM270 | *lys2/lys2* | This study |
| YJM302 | YJM270 | *lys5/lys5* | This study |
| YJM304 | YJM280 | *lys2/lys2* | This study |
| YJM307 | Y55 | *lys5/lys5* | This study |
| YJM320 | Segregant of a clinical isolate | *HO/HO* | [4] |
| YJM326 | Segregant of a clinical isolate | *HO/HO* | [4] |
| YJM339 | Segregant of a clinical isolate | *HO/HO* | [4] |
| YJM376 | YJM326 | *lys2/lys2* | [4] |
| YJM378 | YJM320 | *lys2/lys2* | [4] |
| YJM379 | YJM339 | *lys2/lys2* | [4] |
| YJM380 | YJM339 | *lys5/lys5* | This study |
| YJM385 | YJM145 | *lys5/lys5* | [4] |
| YJM421 | Segregant of a clinical isolate | *HO/HO* | [4] |
| YJM591 | Y55 | *lys2/lys2* | This study |
| YJM627 | Y55 | *HO/HO* | This study |
| YJM791 | YJM145 | *ura3/ura3* | This study |
| YRP008 | YJM421 | *lys2/lys2* | This study |
| YRP009 | YJM421 | *lys5/lys5* | This study |
| YRP061 | YJM421 | *HO/ho∆::Kan* | This study |

S288c background strains are designated as “S”; YJM background strains are designated as “YAG”, “YBN”, “YHS”, “YJM”, “YRP”; hybrids generated by crossing S288c background strains with YJM background strains are designated as “XHS”. For XHS hybrid strains of the 10 genetic backgrounds (other than YJM145/S288c hybrids), instead of stating the strains crossed to derive the hybrid strain, only the relevant parental backgrounds are mentioned for simplicity. Unless stated otherwise, the strains are *MATa/MATα* diploids. Asterisk (*) denotes allele replacement genotype of a strain.

**REFERENCES:**

1. Steinmetz LM, Sinha H, Richards DR, Spiegelman JI, Oefner PJ, et al. (2002) Dissecting the architecture of a quantitative trait locus in yeast. Nature 416: 326-330.

2. Ito-Harashima S, McCusker JH (2004) Positive and negative selection *LYS5*MX gene replacement cassettes for use in *Saccharomyces cerevisiae*. Yeast 21: 53-61.

3. Goldstein AL, McCusker JH (1999) Three new dominant drug resistance cassettes for gene disruption in *Saccharomyces cerevisiae*. Yeast 15: 1541-1553.

4. McCusker JH, Clemons KV, Stevens DA, Davis RW (1994) Genetic characterization of pathogenic *Saccharomyces cerevisiae* isolates. Genetics 136: 1261-1269.
