# Supplementary material for: Complex Genetic Interactions in a Quantitative Trait Locus
Source: PLoS Genet. 2006 Feb 3;2(2):e13. doi: 10.1371/journal.pgen.0020013 (PMC1359075; doi:10.1371/journal.pgen.0020013)
Supplement: Supplementary file 5 [file pgen.0020013.st005.doc]

**Table S5:** List of oligonucleotides and PCR primers used in this study.

| **Oligo** | **Comments** | **Sequence (5’ to 3’)** |
| --- | --- | --- |
| BN072 | Verification primer for MX4 Tef promoter, anti-sense orientation | ACGAGGCAAGCTAAACAG |
| BN430 | *RHO2 (*∆256-287 bp) deletion primer, sense orientation | TCCATTCTCATATTCAAAAGCAGATATAATATTAATTGGGcagctgaagcttcgtacgc |
| BN431 | *RHO2 (*∆256-287 bp) deletion primer, anti-sense orientation | ATATCGTAATGCCTCATCCGCCCATTTCGTCCTTGCGgcataggccactagtggatctg |
| BN432 | *RHO2-288* (91F) allele replacement, sense orientation | ATGAACGTTTACGTCCATTCTCATATTCAAAAGCAGATATAATATTAATTGGGTTTGCTGTAGACAATTTTGAATCACTA |
| BN433 | *RHO2-288* (91F) allele replacement, anti-sense orientation | ATTGGTGCGTCAGGACAATATCGTAATGCCTCATCCGCCCATTTCGTCCTTGCGTTAATTAGTGATTCAAAATTGTCTAC |
| BN434 | *RHO2-145* (91C) allele replacement, sense orientation | ATGAACGTTTACGTCCATTCTCATATTCAAAAGCAGATATAATATTAATTGGGTTTGCTGTAGACAATTGTGAATCACTA |
| BN435 | *RHO2-145* (91C) allele replacement, anti-sense orientation | ATTGGTGCGTCAGGACAATATCGTAATGCCTCATCCGCCCATTTCGTCCTTGCGTTAATTAGTGATTCACAATTGTCTAC |
| BN436 | *RHO2* verification primer (-321-302 bp upstream start site), sense orientation | GGTTTTTCTTACCGTCGTGG |
| BN437 | *RHO2* verification primer (+931-949 bp upstream start site), anti-sense orientation | TCTTTCACTCATCGCCTCG |
| BN568 | *RHO2* verification primer (+397-416 bp downstream start site), anti-sense orientation | GGAACCATTTCATCGGTAGC |
| BN710 | *RHO2* 3’UTR deletion, anti-sense orientation | AAAGACGCGTCCTACTGGCGCGTCTTTCACTCATCGCCTCGcagctgaagcttcgtacgc |
| BN711 | *RHO2* 3’UTR deletion, sense orientation | ATGAAGAAGGAACCAGGGGCTAACTGTTGCATAATTTTgcataggccactagtggatctg |
| HS003 | *END3* ORF deletion primer, sense orientation | TATTGGAAAGGCCGGTAAAGATAACAGGGATCTCTGAAAAcagctgaagcttcgtacgc |
| HS005 | *MKT1* ORF deletion primer, sense orientation | TAAAGTACTAAAGGCAGAAAAATTAATAGCAAATTAAGCGcagctgaagcttcgtacgc |
| HS015 | *MKT1* verification primer (-364-345 bp upstream start site), sense orientation | ATCTGGAAAGCAAGAACGTA |
| HS016 | *MKT1* verification primer (+2621-2641 bp downstream start site), anti-sense orientation | GTTGCTATTCACTGTGCTTGT |
| HS017 | *END3* verification primer (-111-92 bp upstream start site), sense orientation | GAACGTCAGAAACACAAGGT |
| HS018 | *END3* verification primer (+1463-1483 bp downstream start site), anti-sense orientation | CTGAAGCGTGAGAATGAGTAT |
| HS022 | *END3* ORF deletion primer, anti-sense orientation | TATTACACATTCATGTACATAAAATTAATTATCGGTGgcataggccactagtggatctg |
| HS023 | *MKT1* ORF deletion primer, anti-sense orientation | ATAGTTCCACTATTTCCATCATACTCATTCTCACGCTgcataggccactagtggatctg |
| HS098 | *END3* deletion verification primer (+1362-1383 bp downstream stop site) with JM37, anti-sense orientation | GAGGGTTCAAGTACCATCATCA |
| HS099 | *MKT1* deletion verification primer (+2776-2795 bp downstream start site) with JM37, anti-sense orientation | CTTCTCTGCCGGAATTTCAT |
| HS155 | *MKT1* verification primer (+1-20 bp downstream start site), sense orientation | ATGGCAATCAAGTCATTGGA |
| HS156 | *MKT1* verification primer (+1479-1498 bp downstream start site), anti-sense orientation | TGGTCAAAGTGGTAGGCTCA |
| HS157 | *END3* verification primer (+532-551 bp downstream start site), sense orientation | AACCCACAAAATTTGCCATC |
| HS158 | *END3* verification primer (+1010-1029 bp downstream start site), anti-sense orientation | TGCTTGCAATTCGTGTCTCT |
| HS159 | *ARP2* ORF deletion primer, sense orientation | ACAAATCACAGTAATAAACTAACAAAACTATATAATAATAcagctgaagcttcgtacgc |
| HS160 | *ARP2* ORF deletion primer, anti-sense orientation | TTTTTTATTAATTCTTTTGGATCAATAAAGTTCAAATgcataggccactagtggatctg |
| HS161 | *ARP2* deletion verification primer (+1738-1757 bp downstream start site) with JM37, anti-sense orientation | TGCCGTTAGATATGGCTCTG |
| HS162 | *PAN1* ORF deletion primer, sense orientation | TCTATCAGCGACGCGAAATTGCTAATCTGTACAACTGAATcagctgaagcttcgtacgc |
| HS163 | *PAN1* ORF deletion primer, anti-sense orientation | AGTATACATACGTATCTATAGAAAGCAAATTAAATCTgcataggccactagtggatctg |
| HS164 | *PAN1* deletion verification primer (+4815-4834 bp downstream start site) with JM37, anti-sense orientation | AGCAGGGATGAAAACACAGG |
| HS167 | *MKT1* deletion verification primer  (-471-451 bp upstream start site) with BN072, sense orientation | TAGCATAATCTTCGATTCCTT |
| HS168 | *MKT1-END3* primer for cloning upstream fragment (-389-360 bp upstream *YNL086W* start site), sense orientation | TCCACCGCGGTGGCGGCCGCTCTAGAACTATAATTCTATCGATGGTAAAGAGTATGTGTC |
| HS169 | *MKT1-END3* primer for cloning upstream fragment (-501-472 bp upstream *MKT1* start site), anti-sense orientation | TACATGTGTATGTTTACTTAGTTGTTGAGG |
| HS170 | *MKT1-END3* primer for cloning downstream fragment (-575-547 bp upstream *END3* start site), sense orientation | GATAGATTTAGATCATGACGGTAAGATTG |
| HS171 | *MKT1-END3* primer for cloning downstream fragment (+837-865 bp downstream *YNL083W* start site), anti-sense orientation | GTCGACGGTATCGATAAGCTTGATATCGAACTAATGGAGATGATATCTTGTTTATGTCA |
| HS177 | *RHO2* ORF deletion primer, sense orientation | CTGAAACGTTCTGCTTTGGTTGTGCTTTTGATCCCGTACTcagctgaagcttcgtacgc |
| HS178 | *RHO2* ORF deletion primer, anti-sense orientation | CTAAAAAGATAATGTATCATTTCAGTGTAAGTTTTTTgcataggccactagtggatctg |
| HS181 | *RHO2* deletion verification primer (+924-943 bp downstream start site) with JM37, anti-sense orientation | ACTCATCGCCTCGTAAGGAA |
| HS203 | *RHO2* primer for cloning upstream fragment (+309-337 bp downstream *YNL092W* start site), sense orientation | TCCACCGCGGTGGCGGCCGCTCTAGAACTACTCTTTACTTACGCAAGTTTATAGGGAGT |
| HS204 | *RHO2* primer for cloning upstream fragment (+980-1009 bp downstream *YNL092W* start site), anti-sense orientation | GTGTATAGTCATTGATTTTATCTTGAAACC |
| HS205 | *RHO2* primer for cloning downstream fragment (+269-298 bp downstream *TOP2* start site), sense orientation | CTGAGGAACATACTATAGAAGTGAAAAATG |
| HS206 | *RHO2* primer for cloning downstream fragment (+880-909 bp downstream *TOP2* start site), anti-sense orientation | GTCGACGGTATCGATAAGCTTGATATCGAAAGAAATTTGTTGAAAAGAGATATCAGAAAC |
| HS207 | *END3* deletion verification primer  (-515-496 bp upstream start site) with BN072, sense orientation | CATTGGAAGCATACTTCTTG |
| HS208 | *RHO2* deletion verification primer  (-522-503 bp upstream start site) with BN072, sense orientation | TCCAACTGCTACACCATTTA |
| HS233 | *END3* (∆773-802 bp) deletion primer, sense orientation | GACAATCTGCGTGACAATGGGCAGAACTCGAGTTCCGATAcagctgaagcttcgtacgc |
| HS234 | *END3* (∆773-802 bp) deletion primer, anti-sense orientation | TCATCTGTCAGAGAAGCATATTTTTGCTTTATACTGTgcataggccactagtggatctg |
| HS235 | *END3-145(258N)* allele replacement, sense orientation | TAACGAAGTGGACAATCTGCGTGACAATGGGCAGAACTCGAGTTCCGATAGCAGTGGCAGTAACGTGCTTTCAAATGAAA |
| HS236 | *END3-145(258N)* allele replacement, anti-sense orientation | GTTCGCAACTTGATCATCTGTCAGAGAAGCATATTTTTGCTTTATACTGTTTTCATTTGAAAGCACGTTACTGCCACTGC |
| HS237 | *END3-145(268N)* allele replacement, sense orientation | TAACGAAGTGGACAATCTGCGTGACAATGGGCAGAACTCGAGTTCCGATAACAGTGGCAGTAACGTGCTTTCAAATGAAG |
| HS238 | *END3-145(268N)* allele replacement, sense orientation | GTTCGCAACTTGATCATCTGTCAGAGAAGCATATTTTTGCTTTATACTGTCTTCATTTGAAAGCACGTTACTGCCACTGT |
| HS239 | *END3-145(258N 268N)* allele replacement, sense orientation | TAACGAAGTGGACAATCTGCGTGACAATGGGCAGAACTCGAGTTCCGATAGCAGTGGCAGTAACGTGCTTTCAAATGAAG |
| HS240 | *END3-145(258N 268N)* allele replacement, anti-sense orientation | GTTCGCAACTTGATCATCTGTCAGAGAAGCATATTTTTGCTTTATACTGTCTTCATTTGAAAGCACGTTACTGCCACTGC |
| HS241 | *END3-288(258S)* allele replacement, sense orientation | TAACGAAGTGGACAATCTGCGTGACAATGGGCAGAACTCGAGTTCCGATAACAGTGGCAGTAACGTGCTTTCAAATGAAG |
| HS242 | *END3-288(258S)* allele replacement, anti-sense orientation | GTTCGCAACTTGATCATCTGTCAGAGAAGCATATTTTTGCTTTATACTGTCTTCATTTGAAAGCACGTTACTGCCACTGT |
| HS243 | *END3-288(268D)* allele replacement, sense orientation | TAACGAAGTGGACAATCTGCGTGACAATGGGCAGAACTCGAGTTCCGATAGCAGTGGCAGTAACGTGCTTTCAAATGAAA |
| HS244 | *END3-288(268D)* allele replacement, anti-sense orientation | GTTCGCAACTTGATCATCTGTCAGAGAAGCATATTTTTGCTTTATACTGTTTTCATTTGAAAGCACGTTACTGCCACTGC |
| HS245 | *END3-288(258S 268D)* allele replacement, sense orientation | TAACGAAGTGGACAATCTGCGTGACAATGGGCAGAACTCGAGTTCCGATAACAGTGGCAGTAACGTGCTTTCAAATGAAA |
| HS246 | *END3-288(258S 268D)* allele replacement, anti-sense orientation | GTTCGCAACTTGATCATCTGTCAGAGAAGCATATTTTTGCTTTATACTGTTTTCATTTGAAAGCACGTTACTGCCACTGT |
| HS247 | *END3* (∆773-802 bp) deletion verification primer (+1362-1384 bp downstream start site) with JM37, anti-sense orientation | AGAGGGTTCAAGTACCATCATCA |
| HS248 | *END3* (∆773-802 bp) deletion verification primer (-439-419 bp upstream start site) with BN072, sense orientation | TTTGATTGCCTCATCATTTCC |
| HS251 | *MKT1* (∆69-110 bp) deletion primer, sense orientation | TTTTCGAAAGAGGTCTAGTAGGATCCTATGCCATTGAGGCcagctgaagcttcgtacgc |
| HS252 | *MKT1* (∆69-110 bp) deletion primer, anti-sense orientation | CAAATACTGTTCTCTTTTATTGGTCAACAATCTGGAAgcataggccactagtggatctg |
| HS253 | *MKT1* (∆1339-1379 bp) deletion primer, sense orientation | TGTCACAGGAGTTTATCCTGAAGAGCCTCCATTGGGCGGAcagctgaagcttcgtacgc |
| HS254 | *MKT1* (∆1339-1379 bp) deletion primer, anti-sense orientation | AGTTAATAAATTGATTTCTTTATTCTTGAAGATTTCAgcataggccactagtggatctg |
| HS255 | *MKT1* (∆69-110 bp) deletion verification primer (+455-474 bp downstream start site) with JM37, anti-sense orientation | TGGCTCTTGGGGTTGAATAG |
| HS256 | *MKT1* (∆1339-1379 bp) deletion verification primer (+905-925 bp downstream start site) with BN072, sense orientation | CAACCTTACAAAATGACTCCA |
| HS257 | *MKT1* (∆1339-1379 bp) deletion verification primer (+1661-1680 bp downstream start site) with JM37, anti-sense orientation | TTTGGATTCGATTGGTACGG |
| HS258 | *MKT1-145(30G)* allele replacement, sense orientation | TTTTCGAAAGAGGTCTAGTAGGATCCTATGCCATTGAGGCTCTGAATAATTGTACCCTGGGTATAGACGTCAACCATTAT |
| HS259 | *MKT1-145(30G)* allele replacement, anti-sense orientation | TCCAAATACTGTTCTCTTTTATTGGTCAACAATCTGGAAACATAATGGTTGACGTCTATACCCAGGGTACAATTATTCAG |
| HS260 | *MKT1-288(30D)* allele replacement, sense orientation | TTTTCGAAAGAGGTCTAGTAGGATCCTATGCCATTGAGGCTCTGAATAATTGTACCCTGGATATAGACGTCAACCATTAT |
| HS261 | *MKT1-288(30D)* allele replacement, anti-sense orientation | TCCAAATACTGTTCTCTTTTATTGGTCAACAATCTGGAAACATAATGGTTGACGTCTATATCCAGGGTACAATTATTCAG |
| HS262 | *MKT1-145(453R)* allele replacement, sense orientation | TTGTCACAGGAGTTTATCCTGAAGAGCCTCCATTGGGCGGAGGATCTTCTACATCCTACAGGAAACTGGTTTCCAAGTCC |
| HS263 | *MKT1-145(453R)* allele replacement, anti-sense orientation | TGAGTTAATAAATTGATTTCTTTATTCTTGAAGATTTCAACGGACTTGGAAACCAGTTTCCTGTAGGATGTAGAAGATCC |
| HS264 | *MKT1-288(453K)* allele replacement, sense orientation | TTGTCACAGGAGTTTATCCTGAAGAGCCTCCATTGGGCGGAGGATCTTCTACATCCTACAAGAAACTGGTTTCCAAGTCC |
| HS265 | *MKT1-288(453K)* allele replacement, anti-sense orientation | TGAGTTAATAAATTGATTTCTTTATTCTTGAAGATTTCAACGGACTTGGAAACCAGTTTCTTGTAGGATGTAGAAGATCC |
| HS395 | *APM3* ORF deletion primer, sense orientation | ACAACAGAGGCTGTAAACCTTACCAACCCAACCAAAATAGcagctgaagcttcgtacgc |
| HS396 | *APM3* ORF deletion primer, anti-sense orientation | TCTCATTATATTCTATTTAGTTTCGCATGGAATTTCAgcataggccactagtggatctg |
| HS397 | *APM3* deletion verification primer (+1765-1784 bp downstream start site) with JM37, anti-sense orientation | TTTGACTGTTTCCCCTCCTG |
| HS398 | *APM3* deletion verification primer  (-479-460 bp upstream start site) with BN072, sense orientation | ATTAGCAATGGGTTGAGATT |
| HS399 | *RVS161* ORF deletion primer, sense orientation | CAAATCCTATTATAAGAAGCCAGAAGAAAGCTGATACAAGcagctgaagcttcgtacgc |
| HS400 | *RVS161* ORF deletion primer, anti-sense orientation | AATGAAAGAAACATAAATGACCGTAAAAAACTAAAGGgcataggccactagtggatctg |
| HS401 | *RVS161* deletion verification primer (+1163-1182 bp downstream start site) with JM37, anti-sense orientation | CTTTGTTATCCGGCATTCGT |
| HS402 | *RVS161* deletion verification primer (-474-455 bp upstream start site) with BN072, sense orientation | ATGATGTAAGCAAGGTACGG |
| HS403 | *RVS167* ORF deletion primer, sense orientation | GGTCATTTAACACCAAGAATCAAGGAGCCAATAAGTGCACcagctgaagcttcgtacgc |
| HS404 | *RVS167* ORF deletion primer, anti-sense orientation | AACATCTCACAATAGAAGGTAATGAATACAGAGGGATgcataggccactagtggatctg |
| HS405 | *RVS167* deletion verification primer (+1847-1866 bp downstream start site) with JM37, anti-sense orientation | CCAGGCCACTAGTCTTGCTT |
| HS406 | *RVS167* deletion verification primer (-427-408 bp upstream start site) with BN072, sense orientation | TGTTACCTGGGAGATAATGG |
| HS415 | *PBP1* ORF deletion primer, sense orientation | TCACGGAAGTAATTAAAGGAGTTCATTACATTGAAACAATcagctgaagcttcgtacgc |
| HS416 | *PBP1* ORF deletion primer, anti-sense orientation | AGTGCATGAATTTATTACTATATATTGCTTTTTCTGAgcataggccactagtggatctg |
| HS417 | *PBP1* deletion verification primer (+2487-2506 bp downstream start site) with JM37, anti-sense orientation | ATCGGTGGCCTACAGAGTTC |
| HS418 | *PBP1* deletion verification primer  (-491-472 bp upstream start site) with BN072, sense orientation | ACCAAGATAAGACCGAAGAA |
| JM37 | Verification primer for MX4 Tef terminator, sense orientation | CCTCGACATCATCTGCCC |
| JM41 | 3’ tag sequence of sense orientation deletion primer with homology to MX4 cassette | cagctgaagcttcgtacgc |
| JM42 | 3’ tag sequence of anti-sense orientation deletion primer with homology to MX4 cassette | gcataggccactagtggatctg |

MX4 drug marker cassette homology (primers JM41 and JM42) in ORF deletion primers is shown in small case and pRS316 [1] plasmid homology in cloning primers is shown as underlined.

**ReferenceS:**

1. Sikorski RS, Hieter P (1989) A system of shuttle vectors and yeast host strains designed for efficient manipulation of DNA in *Saccharomyces cerevisiae*. Genetics 122: 19-27.
